# A simple yet accurate correction for winner's curse can predict signals discovered in much larger genome scans

**DOI:** 10.1093/bioinformatics/btw303

**Published:** 2016-05-13

**Authors:** T. Bernard Bigdeli, Donghyung Lee, Bradley Todd Webb, Brien P. Riley, Vladimir I. Vladimirov, Ayman H. Fanous, Kenneth S. Kendler, Silviu-Alin Bacanu

**Affiliations:** ^1^Department of Psychiatry, Virginia Institute for Psychiatric and Behavioral Genetics; ^2^Center for Biomarker Research & Personalized Medicine, Virginia Commonwealth University, Richmond, VA 23298, USA; ^3^Lieber Institute for Brain Development, Johns Hopkins University, Baltimore, MD 21205, USA

## Abstract

**Motivation:** For genetic studies, statistically significant variants explain far less trait variance than ‘sub-threshold’ association signals. To dimension follow-up studies, researchers need to accurately estimate ‘true’ effect sizes at each SNP, e.g. the true mean of odds ratios (ORs)/regression coefficients (RRs) or *Z*-score noncentralities. Naïve estimates of effect sizes incur winner’s curse biases, which are reduced only by laborious winner’s curse adjustments (WCAs). Given that *Z*-scores estimates can be theoretically translated on other scales, we propose a simple method to compute WCA for *Z*-scores, i.e. their true means/noncentralities.

**Results:**WCA of *Z*-scores shrinks these towards zero while, on *P*-value scale, multiple testing adjustment (MTA) shrinks *P*-values toward one, which corresponds to the zero *Z*-score value. Thus, WCA on *Z*-scores scale is a proxy for MTA on *P*-value scale. Therefore, to estimate *Z*-score noncentralities for all SNPs in genome scans, we propose **F**DR **I**nverse **Q**uantile **T**ransformation (FIQT). It (i) performs the simpler MTA of *P*-values using FDR and (ii) obtains noncentralities by back-transforming MTA *P*-values on *Z*-score scale. When compared to competitors, realistic simulations suggest that FIQT is more (i) accurate and (ii) computationally efficient by orders of magnitude. Practical application of FIQT to Psychiatric Genetic Consortium schizophrenia cohort predicts a non-trivial fraction of sub-threshold signals which become significant in much larger supersamples.

**Conclusions**: FIQT is a simple, yet accurate, WCA method for *Z*-scores (and ORs/RRs, via simple transformations).

**Availability and Implementation:** A 10 lines R function implementation is available at https://github.com/bacanusa/FIQT.

**Contact:** sabacanu@vcu.edu

**Supplementary information:** Supplementary data are available at *Bioinformatics* online.

## 1 Introduction

Genome-wide association studies (GWAS) represent a powerful and widely used tool for detecting associations between genetic variants and complex traits. In such studies, researchers directly assay and statistically impute (Li *et al.*, 2010) genotypes for one to several million single nucleotide polymorphisms (SNPs), respectively. The GWAS paradigm has been very successful in identifying genetic variants associated with a range of phenotypes (Dewan *et al.*, 2006; Hindorff *et al.*, 2009; Wellcome Trust Case Control Consortium, 2007). However, as seen in GWAS of psychiatric disorders (Purcell *et al.*, 2009; Sklar *et al.*, 2011), a considerable portion of the predicted genetic contribution is contributed by numerous moderate signals which are not deemed significant at accepted genome-wide levels, i.e. ‘suggestive’ signals. With the advent of large-scale, whole-exome and -genome sequencing studies, the field will likely see an exponential increase in the number of such suggestive signal.

To successfully dimension future studies, e.g. for detecting as significant a certain number of signals which are only ‘suggestive’ in current cohorts, there is a need for statistical methods that accurately estimate unbiased effect-sizes for suggestive signal SNPs and, even, all variants from genome scans (henceforth denoting not only extant GWAS and whole-exome sequencing but emergent whole-genome sequencing studies as well). Given that effect size estimates such as *Z*-scores, ORs and RRs can be theoretically translated from one scale to another, in this paper we concentrate on estimating the true means/noncentrality of *Z*-scores at each SNP.

When estimating the true effect sizes, it is well established that the largest signals are generally the most affected by the bias known as ‘winner's curse’ (Zollner and Pritchard, 2007), i.e. their apparent effect size is (sometimes much) larger than their true values. This is due to statistics with the largest magnitude having an extreme value distribution (Jenkinson, 1955), as opposed to the Gaussian distribution we commonly assume for a random SNP *Z*-score. By incorrectly assuming a Gaussian distribution, naïve estimators of extreme statistics have a tendency to overestimate the magnitude of these statistics (Zollner and Pritchard, 2007). In statistical genetics, researchers proposed a multitude of methods to perform winner’s curse adjustment (WCA) for studies with one-stage (discovery) (Faye *et al.*, 2011; Ghosh *et al.*, 2008; Sun *et al.*, 2011; Xiao and Boehnke, 2011; Xu *et al.*, 2011; Zhong and Prentice, 2007, 2008;) and two-stage (discovery and replication) studies (Bowden and Dudbridge, 2009; Zhong and Prentice, 2008). For *Z*-scores, these WCA estimates, i.e. their noncentralities or true means, are obtained by shrinking the *Z*-scores towards their null value of zero. However, a majority of these methods are only designed to handle mostly significant signals.

Recently, two new tools for estimating the mean/noncentrality of all statistics in genome scans were proposed. The first, the Empirical Bayes (EB) method based on Tweedie’s formula (Efron, 2009), was adapted from a general purpose statistical method. Because it employs empirical estimates of the density/histogram (120 bins by default) of scan statistics, it is well suited for the large number of statistics from a genome scan (albeit less suited to instances in which the number of statistics is much smaller). In the context of genome scans, this method was used by Ferguson *et al.* (2013), who found that the empirical histogram is less precise in the extreme tails off the distribution, where tail adjustment (TA) methods (Ghosh *et al.*, 2008; Zhong and Prentice, 2008) provide better accuracy. Based on these observations, the authors proposed an interesting adaptive combination of EB and TA which, at the cost of increased computational burden, combines the best attributes of both methods. The second of these new tools is a computationally efficient, soft threshold method (Bacanu and Kendler, 2012) which adjusts statistics such that their sum of squares do not overestimate the true mean. Because this method does not use empirical density estimation, it is applicable even to a small number of statistics.

Similar to WCA, naïve use of SNP *P*-values as a measure of association for SNPs will overestimate their statistical significance. This is due to the fact that, due to the large number of tests, many SNPs will attain very low *P*-values even under the null hypothesis of no association between trait and genotypes. Thus, to be used in assessing genome-wide significance of SNPs, individual *P*-values need to be first adjusted for multiple testing (Benjamini and Hochberg, 1995; Dudbridge and Gusnanto, 2008). After multiple testing adjustment (MTA), the adjusted *P*-values are much larger than the original ones, i.e. they are ‘shrunken’ towards the null value of one. Given that WCA shrinks *Z*-scores towards zero and MTA shrinks the *P*-values towards one (which corresponds to a value of zero on the *Z*-score scale), we argue that WCA for effect sizes is very similar in spirit with, if not downright the homologue of, MTA for *P*-values. Thus, MTA can be considered, if not identical to, a very good proxy for the WCA for *P*-values.

To accurately estimate the WCA of *Z*-scores from a genome scan, i.e. their true means/noncentralities, we propose a novel two-step method, which is inspired by the strong similarity between MTA and WCA. First, we perform a MTA for *P*-values, e.g. by using a False Discovery Rate (FDR) approach (Benjamini and Hochberg, 1995). Second, we estimate the noncentrality of *Z*-scores by back-transforming the adjusted *P*-value on the *Z*-score scale using an inverse Gaussian cumulative distribution function (cdf). When compared to competing methods, we show that the proposed procedure has very good performance in terms of (i) squared error loss, (ii) fraction of the variability in true means of univariate statistics explained and (iii) computational efficiency. A practical application of this approach shows that, due to their good performance, the proposed estimators can be used to predict with reasonable accuracy the number and location of subtreshold signals that are likely to become significant in much larger cohorts.

## 2 Methods

As mentioned above, the main issue we address in this paper pertains to the estimation, as opposed to testing, of *Z*-score noncentralities for all SNPs in a genome scan. Below, we first present our proposed method and its competitors, including extensions of these competitors proposed by us. Subsequently, we describe simulation setup and genetic data used for our chosen practical application.

### 2.1 Notation

Let $X_{i}∼N\left(\mu_{i},1\right),\;i=1,\ldots ,k,$ be the normally distributed univariate statistics from a genome scan and $\mu_{i}$ and $p_{i},\;i=1,\ldots ,k,$ their associated noncentralities and *P*-values, respectively. If not reported, $X_{i}$ can be easily computed based on other reported summary statistics [see Supplementary Material (SM)].

### 2.2 Novel method based on *P*-value adjustment

Given the extreme value distribution of scan statistics in the upper and lower tails and different distributions elsewhere, it is unclear (or, at least, very complicated) how to properly WCA *Z*-scores, i.e. estimate their noncentralities, for all SNPs in a genome scan. However, it is extremely simple to MTA the *P*-values for the genome-wide multiple testing. The Invariance Principle of Mathematical Statistics (Casella and Berger, 1990) implies that a legitimate, *and thus not merely ad-hoc*, statistical approach is to perform the winner’s curse adjustment on any scale/transformed variable and transform the adjusted quantities on the original scale. Based on the Invariance Principle and the argument in the Introduction, stating that MTA is a (very) good proxy for WCA on the *P*-value scale, we thus propose to (i) adjust SNP *P*-values for multiple testing, e.g. using FDR as in this paper and (ii) obtain the *Z*-score noncentrality estimates for each SNP as the Gaussian quantiles (with the appropriate sign) associated with these adjusted *P*-values (also denoted as FDR *q*-values). [While the FDR procedure might be anticonservative for the extreme scenario of numerous negatively correlated variables (Benjamini and Hochberg, 1995), in genetics FDR is widely used because (i) *Z*-scores are only locally correlated and (ii) we do not expect these local correlations to be mostly negative.]

In mathematical notation, let ${p_{i}}^{*},i=1,\ldots ,k$ be the *q*-values (FDR adjusted *P*-values). Then, we can estimate the noncentralitiy of *Z*-scores, $\hat{{X_{i}}^{*}},i=1,\ldots ,k$, by

$\hat{{X_{i}}^{*}}=sign\left(X_{i}\right){\;\Phi }^{-1}(1-\frac{{p_{i}}^{*}}{2})$, where ϕ is the cdf of a Gaussian distribution.

While we chose FDR as a simple, and less conservative than most, *P*-value adjustments for multiple testing, homologous methods can be constructed using other *P*-value MTA methods. Many such adjustments are already available in the same *p.adjust* R function, which we employed for the FDR adjustment. Among others, *p.adjust* implements more conservative family-wise error rate type of adjustments, such as Bonferroni, ${p_{i}}^{*}=max(kp_{i},1)$, or Holm’s (1979). While we believe that methods based on the above MTA methods will provide rather similar mean *Z*-score estimates, the assessment of non-FDR based methods are outside the scope of this paper.

### 2.3 EB extensions

EB uses all genome scan statistics to (i) empirically estimate their density and (ii) use the derivatives of the empirical densities to estimate the mean of the statistics and their variance. However, scan statistics are often rather correlated locally (i.e. as a consequence of linkage disequilibrium). This is likely to (i) affect the density estimate (which assumes independent statistics) and (ii) underestimate the variance of mean statistics. To eliminate (most of) the local correlations we propose an EB extension which (i) divides the statistics into *n* equally spaced non-overlapping sets (e.g. first set contains statistics with indices 1,n+1, 2n+1,…, and the second those with indices 2,n+2, 2n+2,…,), (ii) estimates the density for each set, (iii) uses each set density to estimate a set-specific noncentralities for all scan statistics and (iv) estimates the overall noncentralities of scan statistics as the average set-specific means. We denote this estimator as EB-*n*, i.e. when using 100 non-overlapping sets the EB extension is denoted as EB-100. The obvious disadvantage of EB-*n* over EB is its increased computational burden, as the computationally intensive estimation of density and its derivatives are computed n times.

### 2.4 Methods used for comparison

For comparison, we use the naïve maximum likelihood estimator (MLE), i.e. the statistics themselves, classical EB (EB-1 in the above set notation) and EB-*n* (*n *=* *10, 50, 100). (Because the soft threshold method (Bacanu and Kendler, 2012) was found to slightly underperform EB-1, for brevity, we omit it from our results.) Due to it sometimes outperforming EB in the tails, we also include the tail adjustment (TA) method (Ghosh *et al.*, 2008; Zhong and Prentice, 2008). The original TA adjusts all statistics above a preset (and generally significant) threshold, which results in two unusual features for our presentation of results. First, given that remaining methods adjust all statistics in a genome scan, we employed TA outside its intended purposes, e.g. even for (very) non-significant thresholds. Second, given TA’s approach of computing the bias for all statistics above a signal threshold, we present the performance of tested methods (MSE and *R*^2^ in Section 3) in a cumulative manner, i.e. for all statistics with unadjusted *P*-values below a large range of thresholds.

### 2.5 Implementation and assessment of performance

We implemented all described methods using the R statistical programming environment. For FDR, FIQT employed the *p.adjust* base function with the *‘fdr’* option specified for method (10-line FIQT implementation in R, which is available in SM). Based on the descriptions from (Efron, 2009; Ferguson *et al.*, 2013), EB type methods were implemented in three steps. First, the range of *Z*-score vector, Z, is divided into120 equally sized bins. Second, we used gam function in gam package to estimate the probability density function, P(z), as the predicted curve from a smoothed (using natural splines) Poisson regression of bin counts on bin midpoints. Third, the *Z*-score means are estimated numerically for each observed value of Z=z as   $\frac{d\left\{\ln\left[P\left(Z\right)\right]\right\}}{dz}$. The running time of tested methods was assessed using the second entry (i.e. *‘system’*) from the output of *system.time* R function.

### 2.6 Simulations

We simulate a complex height-like trait by patterning our simulations on the observed effect sizes of the m=180 significant signals from the large mega-analysis of human height (MHH), which analyzed around 180, 000 subjects (Lango *et al.*, 2010). We assumed that the trait under investigation has $m_{1}$ causal loci which represent a fraction $\gamma_{c}\leq \;1$ of the number of significant loci (m=180) in height study (Table 1), i.e. $m_{1}=\gamma_{c}\;m$. When $\gamma_{c}<1$, the $m_{1}$ causal loci are chosen at random from the significant loci in MHH. Given that simulating genome scan statistics starting from genotypes is laborious and very time consuming, we simulate scan statistics by adding to subsets of above selected (and scaled) effect sizes, and their LD-induced decay in neighboring SNPs, ARMA (3,4) residuals [for more information see Simulation model in Supplementary Material (SM) and Table 1]. This model for residuals was found to be adequate for simulating statistics for markers with a density of approximately 1 SNP/kbp (Bacanu and Kendler, 2012).

**Table 1.** Simulation design parameters. MHH is the abbreviation for mega-analysis of human height

Parameter name	Parameter	Design levels
ARMA(3,4) model	AR vector	{0.8716, 0.9782, -0.851}
	MA vector	{-0.665, -0.998, 0.659, 0.025}
Number of simulated autosome SNPs	k	2,866,105 (1 SNP/Kbp)
Phenotyped sample size (thousands)	$n_{1}$	{22.5, 45, 90, 180, 360} (fraction of MHH sample:$\gamma_{s}=\left\{\frac{1}{8},\;\frac{1}{4},\;\frac{1}{2},\;1,\;2\right\}$)
Number of causal SNPs	$m_{1}$	{6, 11, 23, 45, 90, 180} (fraction of MHH number of causal SNPs: $\gamma_{c}=\left\{0,\frac{1}{32},\frac{1}{16},\frac{1}{8},\;\frac{1}{4},\;\frac{1}{2},\;1\right\}$)

To assess the performance of methods for underpowered studies, we performed simulations under $H_{0}$. Under this scenario $\gamma_{c}=0$, i.e. the simulated statistics are identical to an ARMA (3,4) realization of unit variance. We simulated sample sizes equaling a fraction $\gamma_{s}\in \{\frac{1}{8},2\}$ of the MHH sample size (n≈180,000). [While the chosen sample sizes (>22 500 subjects) might appear too large, these cohorts (i) consist of unselected/population subjects and (ii) are roughly the sizes of the more powerful selected (case–control) subjects needed to detect a non-trivial number of signals in multi-site psychiatric genetic cohorts (Ripke *et al.*, 2013; Ripke *et al.*, 2011; Sklar *et al.*, 2011).] Additional details regarding the relationship between mean of the statistics and $\gamma_{s}$, are available in the Simulation model subsection in SM. For every parameterization given in Table 1, we performed 250 simulations.

### 2.6 Practical application

To underline FIQT accuracy and its usefulness in genetics, we applied it to the summary statistics from the discovery phase of the 2005 Psychiatric Genomics Consortium (PGC) GWAS of schizophrenia (PGC-SCZ1) (Ripke *et al.*, 2011). PGC-SCZ1 FIQT estimates were used to predict the genomic regions harboring statistics which are expected to attain significance in the four-fold larger discovery phase of the 2014 PGC SCZ study (henceforth denoted as PGC-SCZ2) (Schizophrenia Working Group of the Psychiatric Genomics Consortium, 2014). Our inference is a point prediction, as opposed to a testing procedure. It is based solely on the predicted noncentrality estimates in PGC-SCZ2, as opposed to association *P*-values from PGC-SCZ1. The inference relies on the fact that the *Z*-score noncentrality increases with the square root of the sample size. Thus, the noncentrality (true mean) of *Z*-score for SNPs in PGC-SCZ2 are estimated simply as the double of their PGC-SCZ1 homologues. We predict as significant in PGC-SCZ2 only those SNPs for which the *P*-value associated with their predicted *Z*-score noncentrality is lower than the commonly used $5\times 10^{-8}$ threshold.

## 3 Results

Among EB-*n* methods, we tested EB-1, EB-10, EB-50 and EB-100. EB-10 and EB-50 have intermediate performance between EB-1 and EB-100, with EB-10 closer to EB-1 and EB-50 closer to EB-100 (data not shown). Consequently, we present only the results for EB-1 and EB-100. Measures of prediction accuracy for all of the above methods are assessed based on the simulated and estimated noncentralities for all SNPs in genome scans.

**Fig. 1.**
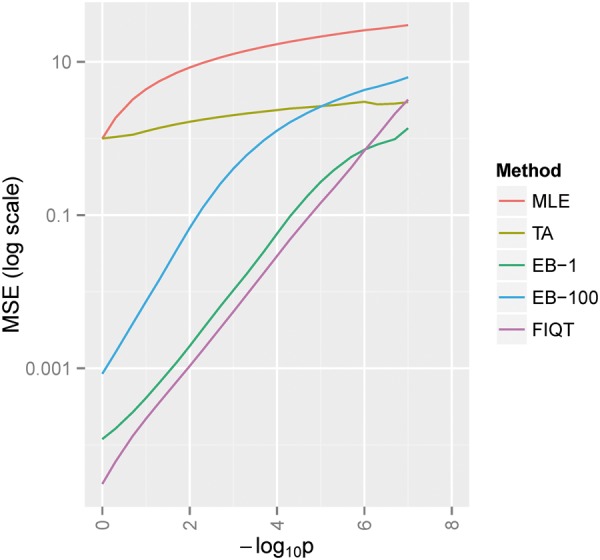
Null hypothesis mean square error (MSE) of *Z*-scores noncentrality estimates for SNPs having negative log unadjusted *P*-values below -log_10_ *P*. Methods abbreviations: MLE – original statistics, TA – tail adjustment, EB-1 – Empirical Bayes, EB-100 – Empirical Bayes 100 subsets and FIQT – our proposed method (FDR Inverse Quantile Transformation)

Under $H_{0}$, i.e. the surrogate for underpowered studies, FIQT has the best mean square error (MSE) performance everywhere, except for the (very small) region of extremely low *P*-values, where EB-1 slightly outperforms it (Fig. 1). Among the remaining methods, EB-1 performs best over the entire parameter space and, as expected, MLE has the largest MSE. We note that, in marked contrast to the alternative hypothesis results that follow, EB-1 thoroughly outperforms EB-100.

**Fig. 2.**
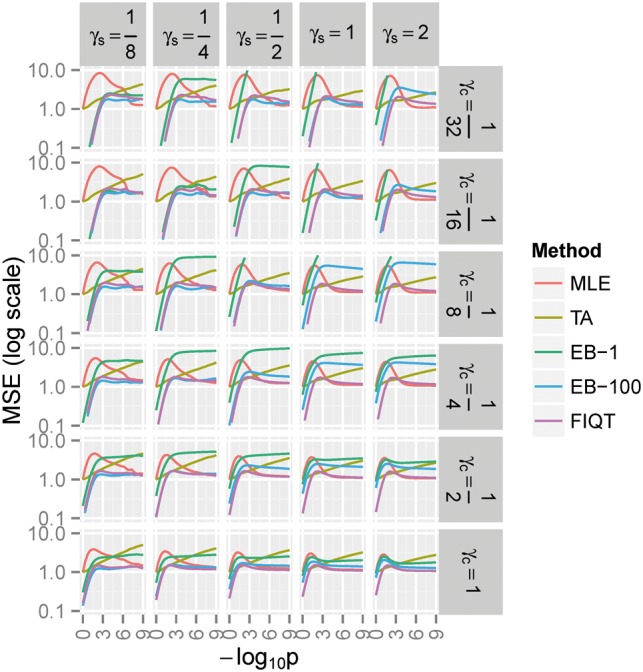
Alternative hypothesis MSE of *Z*-scores noncentrality estimates (see Fig. 1 for background/notation). $\gamma_{s}\;-\;relative\;(to\;MHH)\;sample\;size,\gamma_{c}$ - number of causal signals relative to the 180 significant MHH signals

**Fig. 3.**
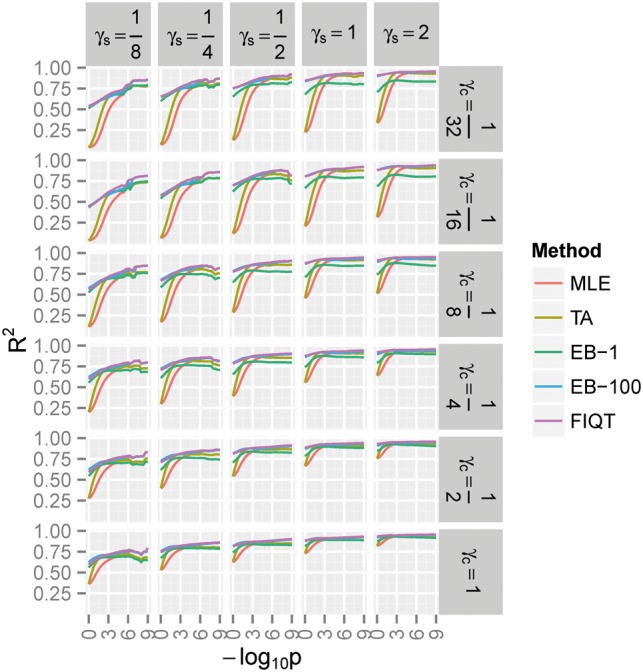
The alternative hypothesis variability (*R*^2^) in *Z*-score noncentrality for SNPs having negative log (unadjusted) *P*-values below -log_10_ *P*. See Figures 1 and 2 for background and notations

Under the alternative, $H_{a}$, FIQT has better MSE performance for settings with moderate to large number of signals and larger sample sizes (Fig. 2). Its performance improvement over competitors is sometimes substantial, e.g. for large sample sizes and medium number of signals. EB-1 does not outperform FIQT under any $H_{a}\;$scenarios. EB-100 only nominally outperforms FIQT at smaller sample sizes. Surprisingly, even though it was designed only as a tail bias adjustment, TA performs reasonably well. Under certain scenarios, e.g. large sample sizes, it outperforms EBs for statistics with nominally significant *P*-values and even slightly outperforms FIQT for a very narrow range of moderately small *P*-values. The better performance of FIQT is mostly due to the lower variance of this estimator [Fig. S1 in Supplementary Material (SM)], because, when compared with EB (and especially TA) methods, the bias is often somewhat larger (Fig. S2). (However, FIQT conservativeness at low *P*-values (negative bias in S2), opens the possibility of future improvement which take into account the local LD of statistics, as alluded in Discussion.) When measuring accuracy by *R*^2^, i.e. the explained variability of the *Z*-score noncentralities, FIQT practically outperforms all other methods (Fig. 3), albeit EB-100 only nominally so.

Due to its very simple computation, FIQT has much faster running times than competitors. When compared to the next most accurate method, EB-100, the proposed method is faster by more than four orders of magnitude [Fig. S2 in Supplementary Material (SM)]. FIQT is also faster than the less accurate EB-1 by almost two orders of magnitude (data not shown).

*Practical application*. Given that discovery phase of PGC-SCZ2 has around four times the sample size of its PGC-SCZ1 homolog, then the *Z*-score noncentrality in PGC-SCZ2 are expected to be twice as large as the PGC-SCZ1 FIQT estimates (see Fig. S4 for relationships between these estimators and PGC-SCZ1 statistics). By computing, under a Gaussian distribution assumption, the *P*-values associated with estimated PCC_SCZ2 noncentralities, we predict 46 regions (Supplementary Table S1) to attain significance in PGC-SCZ2, as opposed to only 11 present in PGC-SCZ1. The 46 regions were obtained by clustering together predicted significant signals within 250 Kb. Of these significant regions, a very high number, 34 (∼75%), overlap the 105 independent chromosomal regions reported by PGC-SCZ2. A total of 18 predicted PGC-SCZ2 regions overlap the extended MHC region (25–33 Mb) on chromosome 6p from the actual PGC-SCZ2 findings, as opposed to only 5 reported by PGC-SCZ1. Of the overall of 34 overlapping regions, 16 are in loci outside MHC regions, as opposed to just 6 reported by PGC-SCZ1.

## 4 Conclusions

We propose a novel approach, FIQT, to extract information from genome scan statistics by estimating noncentrality (true mean) of *Z*-scores by performing a winner’s curse adjustment (WCA) on *Z*-scores. Due to the high degree of similarity between WCA on *Z*-score scale and multiple testing adjustment (MTA) on *P*-value scale, we propose a two stage procedure. First, use FDR for the MTA of *P*-values. Second, transform the adjusted *P*-values to upper tail *Z*-scores and assign to it the sign of the original statistics. When compared to competing methods, FIQT estimators are shown to (i) have a smaller mean squared error loss, (ii) explain a higher proportion of the true means of the statistics and (iii) have substantially faster running times. The practical application to PGC-SCZ1 data show that FIQT estimators are useful for highlighting, with reasonable specificity, genomic regions likely to attain significance only in much larger supersamples.

Empirical Bayes, EB, and similar methods are currently some of the state-of-the-art approaches for accurately estimating the noncentrality of scan statistics. However, all EB methods are computationally and skill intensive and, due to the need of empirically estimating the probability density of statistics, they might not be appropriate when the number of statistics is reasonably small. Our proposed method, FIQT, eliminates these disadvantages while maintaining a similar (to sometimes much better) prediction accuracy. Its performance advantage over EB based methods is especially notable at high sample sizes and a moderate to large number of true signals. [While, the accuracy was assessed only on the *Z*-score scale, Delta Method (a first order Taylor approximation) from statistical theory states that the relative performance of methods should be similar on other scales (Casella and Berger, 1990), e.g. RRs or ORs.]

FIQT practical application to PGC SCZ summary statistics, were used to predict a large fraction of future signals discovered in a four times larger cohort. This underscores its useful in predicting moderately large, even if non-significant, signals. While ranking regions and predicting as possibly significant in the future a set number of them, in practice the decision on the magnitude of such number is both complex and subjective. In contrast, FIQT can help to objectively determine the number of signals that likely to be significant in the future based on the (i) estimated non-centralities and the (ii) increase in sample size. Such estimates can be used to adequately design well-powered follow-up studies.

FIQT estimates the noncentralities for all *Z*-scores in a genome scan. Sometimes, *Z*-scores are not available and the researchers need to estimate them from other summary data (see SM). Conversely, the adjusted *Z*-scores (e.g. FIQT estimates of their noncentrality) can be subsequently used to estimate the adjusted values for the summary statistics of interest. For instance, if summary data contain only log odds ratio, ln⁡(OR), and their standard error,$\;\hat{\sigma }$, then the vector of *Z*-scores is $X=\frac{ln(OR)}{\sigma }$. Subsequently, we can use the adjusted *Z*-scores (FIQT estimates), $X^{*}$, to estimate the adjusted odds ratio, e.g. as ${ln(OR)}^{*}$ = $\hat{\sigma }{\;X}^{*}$(1) or ${ln(OR)}^{*}=\;\frac{X^{*}}{X}ln(OR$). ${ln(OR)}^{*}\;$can be interpreted as the vector of unbiased (winner’s curse corrected) ln⁡(OR). However, it can be argued that the winner’s curse might be due to underestimation of $\hat{\sigma }$, for instance. While adjustment for this scenario is more complex, a helpful idea might be to substitute $\hat{\sigma }\;$in (1) by its average over the (reasonably tight) allele frequency bin which includes the SNP under investigation.

In its present form, FIQT is conservative and provides only point estimates, i.e. it does not compute the standard deviation (SD) of *Z*-score noncentrality estimates. In the future we plan to extend FIQT to be both less conservative and provide such estimates. Heuristically, for each SNP, such a plan might (i), similarly to EB-n, compute FIQT-n mean *Z*-score noncentrality estimates by using only (quasi-independent) SNPs spaced n lags apart and (ii) obtain n-1 estimates of its SNP *Z*-score noncentrality by interpolation using all FIQT-n which do not include this SNP in its support. Subsequently, for each SNP, (i) the *Z*-score noncentrality might be estimated as the mean of its n-1 FIQT-n noncentralities and (ii) the SD of the mean of FIQT-n scores can be estimated assuming that the n-1 FIQT-n Z-scores follow a (circular) AR time series. When compared to the original application to SNPs in high LD, the quasi-independence of SNPs in a FIQT-n set ensures that FDR *q*-values are much less conservative and, in turn, the updated FIQT estimates become less conservative.

FIQT is a very simple yet powerful method. However, in its present form, it is more of a proof-of-concept and we believe it can be further improved. One direction would be to extend FIQT to accurately estimate other variables besides *Z*-score noncentralities. For instance, shrinkage estimators are widely used for correlation/covariance matrices (Daniels and Kass, 2001). Given that the sample correlations are normally distributed with variances dependent only on the sample size, FIQT can be extended to the estimation of correlation matrices. The extension might involve shrinking the magnitudes of correlation matrix entries toward zero.

FIQT has the potential to be used in the personalized genomics, e.g. the prediction of subject level risk based on whole genome data. Methods for predicting subject level risk typically use summary statistics as input, e.g. LDpred extension (http://biorxiv.org/content/early/2015/03/04/015859) of LD score method (Bulik-Sullivan *et al.*, 2015). Thus an increased accuracy of signal estimation used as input might result in more accurate estimates of an individual’s risk/trait mean.

## 5 Software

FIQT is available from GitHub and Supplementary Material. It will also be implemented in DISTMIX (Lee *et al.*, 2015), our group’s direct imputation software for cosmopolitan cohorts (http://dleelab.github.io/distmix/). (DISTMIX imputes the statistics at the unmeasured SNPs based only on the statistics at the measured SNPs and the LD patterns estimated from a cosmopolitan reference panel.)

## Supplementary Material

Supplementary Data
