# Supplementary material for: A simple yet accurate correction for winner's curse can predict signals discovered in much larger genome scans
Source: Bioinformatics. 2016 May 13;32(17):2598–603. doi: 10.1093/bioinformatics/btw303 (PMC5013908; doi:10.1093/bioinformatics/btw303)
Supplement: Supplementary Data [file supp_32_17_2598__index.html]

A simple yet accurate correction for winner's curse can predict signals discovered in much larger genome scans — A simple yet accurate correction for winner's curse can predict signals discovered in much larger genome scans — Supplementary Data 

# A simple yet accurate correction for winner's curse can predict signals discovered in much larger genome scans

## Supplementary Data

files

- Supplementary Data - docx file
