# Supplementary material for: A simple yet accurate correction for winner's curse can predict signals discovered in much larger genome scans
Source: Bioinformatics. 2016 May 13;32(17):2598–603. doi: 10.1093/bioinformatics/btw303 (PMC5013908; doi:10.1093/bioinformatics/btw303)
Supplement: Supplementary Data [file supp_btw303_SM_with_table_in.docx]

**Supplementary Material**

1. **Obtaining normally distributed statistics**

Many GWAS studies report $\text{χ}^{2}$ statistics with one degree of freedom (df) along with odds ratios (or beta regression coefficients) for the reference (tested) alleles. If only p-values are available, these can be transformed into $\text{χ}^{2}$ statistics using the inverse cumulative distribution function of the $\text{χ}^{2}$ statistics with one df. These $\text{χ}^{2}$ statistics need to be converted into normally distributed statistics (Z-scores). This can be accomplished, for instance, by taking i) the absolute value of the Z-score to be the square root of the $\text{χ}^{2}$ statistic and ii) the sign of the Z-score to be a) the sign of reported log odds ratio for binary traits or b) the sign of the reported slope coefficients for quantitative traits.

1. **Simulation model**

While the performance of tested methods should be assessed via simulations based on a real data set, phenotype simulations and univariate statistics calculation can be extremely computationally intensive, even when using fast analysis tools like PLINK (Purcell, Neale, Todd-Brown, Thomas, Ferreira, Bender, Maller, Sklar, de Bakker, Daly et al. 2007). Consequently, we decided to simulate the GWAS statistics directly by i) assuming that the density of SNPs in our GWAS is 1 SNP/Kbp, ii) assuming that SNPs more than 1 Mbp apart are uncorrelated and iii) employing a time series approach similar in spirit to Roeder et al.(Roeder, Bacanu, Wasserman, and Devlin 2006). To do so, we first estimated R^2^ of SNP genotypes as a function of the distance between SNPs using reference Caucasian haplotypes available from Mach(Li, Willer, Ding, Scheet, and Abecasis 2010). Then we determined which Autoregressive Moving Average (ARMA) model better fits i) the estimated average cumulative R^2^ between the genotype of a SNP and the genotypes of SNPs within 1 Mbp and ii) the estimated average R^2^ between genotypes of SNPs situated {1,2,3,...,1000} Kbp apart. We deemed an ARMA (3,4) having an AR vector of {0.8716, 0.9782, -0.851} and an MA vector of {-0.6652, -0.9976, 0.6594, 0.0252} to be the most desirable model. This model provided the best approximation to the estimated average cumulative R^2^ between the statistic at a certain SNP and the statistics at SNPs within 1 Mbp of it, even though it somewhat overestimates the R^2^ between SNPs separated by intermediate distances (~100-300Kbp).

The number, effect size and genomic position of causal loci was modeled on the $m=180$ significant findings from a mega-analysis of human height (Lango, Estrada, Lettre, Berndt, Weedon, Rivadeneira, Willer, Jackson, Vedantam, Raychaudhuri et al. 2010). We assumed that the phenotype under investigation has $m_{1}$ causal loci which represent a fraction $\gamma_{c}=\{0.25, 0.5, 1\}$ of the number of significant loci in height study (Table I), i.e. $m_{1}=\gamma_{c} m$. When $\gamma_{c}<1$, the $m_{1}$ causal loci are chosen at random from the $m=180$ significant loci in the height study. We simulated sample sizes equaling a fraction $\gamma_{s}=\left\{ 0.125, 0.25, 1 \right\}$ of the height meta-analysis sample size $(n\approx180,000)$. A sample size $n_{1}=0.125 n$ is similar to the sample size of the discovery phase of Psychiatric Genetics Consortium (PGC) schizophrenia meta-analysis (Ripke, Sanders, Kendler, Levinson, Sklar, Holmans, Lin, Duan, Ophoff, Andreassen et al. 2011) and a sample size of $n_{2}=0.25 n$ is similar to type 2 diabetes meta-analysis (Voight, Scott, Steinthorsdottir, Morris, Dina, Welch, Zeggini, Huth, Aulchenko, Thorleifsson et al. 2010) (and close to the sample size of both replication and discovery phases of PGC).

With these assumptions we can compute the mean of univariate statistics for each SNP and use them to simulate GWAS univariate statistics. To do so, we denote by $\boldsymbol{\theta}$ the vector of means of univariate statistics at the $m_{1}$ causal loci as estimated in the height meta-analysis paper (Lango, Estrada, Lettre, Berndt, Weedon, Rivadeneira, Willer, Jackson, Vedantam, Raychaudhuri et al. 2010). It follows that the means of univariate statistics at these causal loci for the simulated sample size, $n_{1}\boldsymbol{=}\gamma_{s} n$, is $\boldsymbol{\theta}_{1}\boldsymbol{=}\sqrt{\gamma_{s}}\boldsymbol{\theta}$. Based on the means of univariate statistics at causal loci,$\boldsymbol{\theta}_{1}$**,** and the ARMA LD structure, we use conditional expectation to compute the vector, $\boldsymbol{\mu}$, of means for univariate statistics at SNPs on a chromosome. To ease the computational burden, we set to zero the means of statistics at SNPs more than 1 Mbp away from the closest causal locus. Based on $\boldsymbol{\mu,}$ we simulate, for each chromosome, SNP statistics as $\boldsymbol{X}=\boldsymbol{\mu+\epsilon}$, where $\boldsymbol{\epsilon}$ is the vector of ARMA(3,4) residuals having unit variance.

1. **FIQT R script**

FIQT<-function(z=z, min.p=10^-300){

pvals<-2*pnorm(abs(z),low=F)

### Very low p-values bounded below to be able to compute (inverse) normal cdf for z’s

pvals[pvals<min.p]<- min.p

adj.pvals<-p.adjust(pvals,method="fdr")

mu.z<-sign(z)*qnorm(adj.pvals/2,low=F)

### Do not use the above adjustment for extremely large Z-scores (above around 37…)

### because their p-values were conservatively raised to min.p

mu.z[abs(z)>qnorm(min.p/2,low=F)]<-z[abs(z)>qnorm(min.p/2,low=F)]

mu.z

}

Figure S1. Smoothed standard deviation (SD) of various estimators at various -log10(p-values). See Fig.1 for background and notation.

Figure S2. Smoothed standard deviation (SD) of various estimators at various -log10(p-values). See Fig.1 for background and notation (TA is omitted due to the figure not being cumulative, as those in main manuscript).

Figure S4. Log10 of the quotient between the running times of EB-100 and FIQT. The red vertical line denotes the mean of the distribution.

Z-score

Figure S5. FIQT estimated means for PGC1 (blue) and PGC2 (red). First bisector (black) added for reference.

Reference List

1. Lango, A.H., Estrada, K., Lettre, G., Berndt, S.I., Weedon, M.N., Rivadeneira, F., Willer, C.J., Jackson, A.U., Vedantam, S., Raychaudhuri, S., et al. 2010. Hundreds of variants clustered in genomic loci and biological pathways affect human height. *Nature* **467**:832-838

2. Li, Y., Willer, C.J., Ding, J., Scheet, P., and Abecasis, G.R. 2010. MaCH: using sequence and genotype data to estimate haplotypes and unobserved genotypes. *Genet Epidemiol.* **34**:816-834

3. Purcell, S., Neale, B., Todd-Brown, K., Thomas, L., Ferreira, M.A., Bender, D., Maller, J., Sklar, P., de Bakker, P.I., Daly, M.J., et al. 2007. PLINK: a tool set for whole-genome association and population-based linkage analyses. *Am J Hum.Genet.* **81**:559-575

4. Ripke, S., Sanders, A.R., Kendler, K.S., Levinson, D.F., Sklar, P., Holmans, P.A., Lin, D.Y., Duan, J., Ophoff, R.A., Andreassen, O.A., et al. 2011. Genome-wide association study identifies five new schizophrenia loci. *Nat.Genet.* **43**:969-976

5. Roeder, K., Bacanu, S.A., Wasserman, L., and Devlin, B. 2006. Using linkage genome scans to improve power of association in genome scans. *Am J Hum.Genet.* **78**:243-252

6. Voight, B.F., Scott, L.J., Steinthorsdottir, V., Morris, A.P., Dina, C., Welch, R.P., Zeggini, E., Huth, C., Aulchenko, Y.S., Thorleifsson, G., et al. 2010. Twelve type 2 diabetes susceptibility loci identified through large-scale association analysis. *Nat.Genet.* **42**:579-589

Table S1. The use of PGC1 results for predicting significant signal regions in PGC2. Notation z- PGC1 Z-score, pval- PGC1 p-value, mu - predicted mean of PGC1 scores and mu.pred - predicted mean in PGC2 (it is 2 x mu).

| **rsid** | **chr** | **bp** | **a1** | **a2** | **z** | **pval** | **mu** | **mu.pred** | **signal**  **region #** |
| --- | --- | --- | --- | --- | --- | --- | --- | --- | --- |
| rs11165934 | 1 | 98,417,967 | A | C | **4.695** | 2.66E-06 | **2.787** | **5.574** | 1 |
| rs9887831 | 1 | 98,431,639 | A | G | **-4.812** | 1.50E-06 | **-2.926** | **-5.852** | 1 |
| rs12067700 | 1 | 98,436,232 | T | G | **4.780** | 1.76E-06 | **2.888** | **5.777** | 1 |
| rs12067567 | 1 | 98,441,888 | T | C | **4.781** | 1.75E-06 | **2.889** | **5.779** | 1 |
| rs11165939 | 1 | 98,443,509 | T | C | **-4.822** | 1.42E-06 | **-2.939** | **-5.877** | 1 |
| rs1625579 | 1 | 98,502,934 | T | G | **5.001** | 5.72E-07 | **3.120** | **6.239** | 1 |
| rs2802535 | 1 | 98,508,258 | T | C | **4.991** | 6.01E-07 | **3.112** | **6.224** | 1 |
| rs2660304 | 1 | 98,512,127 | T | G | **4.948** | 7.48E-07 | **3.070** | **6.140** | 1 |
| rs1702292 | 1 | 98,524,960 | A | G | **-4.777** | 1.78E-06 | **-2.886** | **-5.771** | 1 |
| rs1702291 | 1 | 98,526,167 | T | C | **-4.860** | 1.17E-06 | **-2.980** | **-5.960** | 1 |
| rs2660300 | 1 | 98,528,211 | A | C | **-4.820** | 1.43E-06 | **-2.937** | **-5.873** | 1 |
| rs2660299 | 1 | 98,528,452 | T | C | **4.828** | 1.38E-06 | **2.946** | **5.891** | 1 |
| rs1782812 | 1 | 98,547,502 | A | G | **-4.774** | 1.80E-06 | **-2.882** | **-5.765** | 1 |
| rs1198588 | 1 | 98,552,832 | A | T | **-4.996** | 5.86E-07 | **-3.116** | **-6.233** | 1 |
| rs6703335 | 1 | 243,608,967 | A | G | **-4.654** | 3.26E-06 | **-2.735** | **-5.470** | 2 |
| rs17180327 | 2 | 181,016,133 | A | G | **-4.967** | 6.80E-07 | **-3.087** | **-6.174** | 3 |
| rs17662626 | 2 | 193,984,621 | A | G | **4.665** | 3.09E-06 | **2.748** | **5.495** | 4 |
| rs2675968 | 2 | 233,736,244 | T | C | **4.702** | 2.57E-06 | **2.795** | **5.590** | 5 |
| rs13025591 | 2 | 236,795,343 | A | C | **-4.879** | 1.07E-06 | **-3.003** | **-6.007** | 6 |
| rs4663627 | 2 | 236,796,548 | T | C | **4.809** | 1.52E-06 | **2.923** | **5.846** | 6 |
| rs1962550 | 2 | 236,822,569 | C | G | **-4.658** | 3.19E-06 | **-2.740** | **-5.480** | 6 |
| rs2239547 | 3 | 52,855,229 | T | C | **4.730** | 2.25E-06 | **2.832** | **5.665** | 7 |
| rs4687554 | 3 | 52,864,135 | T | C | **4.657** | 3.21E-06 | **2.738** | **5.477** | 7 |
| rs1508411 | 3 | 62,064,517 | T | G | **-4.683** | 2.82E-06 | **-2.772** | **-5.544** | 8 |
| rs11130874 | 3 | 62,064,769 | A | G | **5.191** | 2.09E-07 | **3.275** | **6.550** | 8 |
| rs11715438 | 3 | 62,064,925 | T | C | **-5.184** | 2.17E-07 | **-3.272** | **-6.544** | 8 |
| rs7646226 | 3 | 62,070,296 | A | C | **5.001** | 5.70E-07 | **3.120** | **6.239** | 8 |
| rs9870965 | 3 | 62,073,316 | A | C | **-4.715** | 2.42E-06 | **-2.812** | **-5.623** | 8 |
| rs17065991 | 3 | 62,076,076 | A | G | **-4.678** | 2.90E-06 | **-2.764** | **-5.529** | 8 |
| rs191558 | 3 | 62,079,016 | A | G | **-5.111** | 3.20E-07 | **-3.223** | **-6.446** | 8 |
| rs349158 | 3 | 62,079,245 | T | C | **5.076** | 3.86E-07 | **3.187** | **6.373** | 8 |
| rs349156 | 3 | 62,079,827 | T | C | **-5.082** | 3.73E-07 | **-3.192** | **-6.384** | 8 |
| rs9838229 | 3 | 180,533,251 | A | C | **4.836** | 1.33E-06 | **2.953** | **5.906** | 9 |
| rs1879248 | 3 | 180,551,214 | T | C | **4.844** | 1.27E-06 | **2.961** | **5.922** | 9 |
| rs1433019 | 5 | 171,984,913 | A | C | **4.665** | 3.09E-06 | **2.748** | **5.495** | 10 |
| rs9379780 | 6 | 25,707,171 | A | G | **5.122** | 3.02E-07 | **3.231** | **6.462** | 11 |
| rs4712969 | 6 | 25,764,192 | A | G | **-4.827** | 1.38E-06 | **-2.945** | **-5.890** | 11 |
| rs1892250 | 6 | 25,769,024 | T | C | **-4.891** | 1.00E-06 | **-3.017** | **-6.034** | 11 |
| rs2328893 | 6 | 25,770,239 | A | G | **-5.018** | 5.21E-07 | **-3.133** | **-6.265** | 11 |
| rs1892253 | 6 | 25,782,314 | T | G | **-4.729** | 2.26E-06 | **-2.832** | **-5.664** | 11 |
| rs1324082 | 6 | 25,801,971 | T | C | **-4.721** | 2.35E-06 | **-2.820** | **-5.639** | 11 |
| rs12182983 | 6 | 25,818,755 | A | G | **-4.904** | 9.39E-07 | **-3.029** | **-6.059** | 11 |
| rs6913879 | 6 | 25,820,428 | T | C | **4.661** | 3.14E-06 | **2.743** | **5.486** | 11 |
| rs6940698 | 6 | 25,821,580 | T | C | **-5.119** | 3.08E-07 | **-3.228** | **-6.456** | 11 |
| rs9461219 | 6 | 25,836,927 | C | G | **5.069** | 4.00E-07 | **3.183** | **6.367** | 11 |
| rs1165148 | 6 | 25,844,710 | T | G | **-4.794** | 1.63E-06 | **-2.906** | **-5.812** | 11 |
| rs1165189 | 6 | 25,849,779 | A | C | **4.790** | 1.67E-06 | **2.901** | **5.802** | 11 |
| rs1182814 | 6 | 25,859,554 | T | C | **4.761** | 1.93E-06 | **2.868** | **5.736** | 11 |
| rs1165164 | 6 | 25,863,481 | A | G | **-4.671** | 3.00E-06 | **-2.755** | **-5.511** | 11 |
| rs1165162 | 6 | 25,863,605 | T | C | **-4.711** | 2.47E-06 | **-2.807** | **-5.614** | 11 |
| rs1165158 | 6 | 25,864,898 | A | C | **-4.680** | 2.87E-06 | **-2.767** | **-5.533** | 11 |
| rs13198474 | 6 | 25,874,423 | A | G | **-4.910** | 9.13E-07 | **-3.034** | **-6.068** | 11 |
| rs13220395 | 6 | 26,055,368 | A | G | **-4.756** | 1.98E-06 | **-2.864** | **-5.727** | 12 |
| rs16891264 | 6 | 26,072,445 | T | C | **4.697** | 2.64E-06 | **2.788** | **5.577** | 12 |
| rs2071303 | 6 | 26,091,336 | T | C | **4.989** | 6.06E-07 | **3.110** | **6.220** | 12 |
| rs1572982 | 6 | 26,094,367 | A | G | **-4.887** | 1.03E-06 | **-3.013** | **-6.026** | 12 |
| rs6918586 | 6 | 26,097,384 | T | C | **4.953** | 7.31E-07 | **3.074** | **6.148** | 12 |
| rs198856 | 6 | 26,102,708 | A | G | **4.996** | 5.86E-07 | **3.116** | **6.233** | 12 |
| rs198854 | 6 | 26,104,057 | T | C | **-5.031** | 4.88E-07 | **-3.145** | **-6.290** | 12 |
| rs198844 | 6 | 26,108,282 | C | G | **-4.918** | 8.76E-07 | **-3.042** | **-6.085** | 12 |
| rs198838 | 6 | 26,113,340 | T | C | **-4.956** | 7.18E-07 | **-3.077** | **-6.154** | 12 |
| rs198837 | 6 | 26,113,398 | A | T | **4.942** | 7.72E-07 | **3.066** | **6.131** | 12 |
| rs13161 | 6 | 26,114,702 | T | C | **4.944** | 7.64E-07 | **3.068** | **6.136** | 12 |
| rs198831 | 6 | 26,116,996 | T | C | **-5.033** | 4.83E-07 | **-3.147** | **-6.294** | 12 |
| rs198829 | 6 | 26,118,893 | A | G | **-5.021** | 5.15E-07 | **-3.135** | **-6.270** | 12 |
| rs198828 | 6 | 26,119,459 | A | G | **5.002** | 5.68E-07 | **3.120** | **6.239** | 12 |
| rs198826 | 6 | 26,121,153 | T | C | **4.946** | 7.56E-07 | **3.069** | **6.137** | 12 |
| rs198825 | 6 | 26,122,502 | A | G | **-4.920** | 8.64E-07 | **-3.045** | **-6.090** | 12 |
| rs198821 | 6 | 26,123,629 | T | C | **-4.968** | 6.78E-07 | **-3.087** | **-6.174** | 12 |
| rs198819 | 6 | 26,124,430 | T | C | **5.022** | 5.10E-07 | **3.136** | **6.273** | 12 |
| rs198817 | 6 | 26,126,524 | A | G | **-5.086** | 3.66E-07 | **-3.197** | **-6.394** | 12 |
| rs198816 | 6 | 26,127,184 | T | C | **5.011** | 5.42E-07 | **3.124** | **6.249** | 12 |
| rs198811 | 6 | 26,128,446 | T | C | **4.999** | 5.77E-07 | **3.119** | **6.238** | 12 |
| rs198809 | 6 | 26,128,766 | A | G | **5.017** | 5.25E-07 | **3.132** | **6.265** | 12 |
| rs198806 | 6 | 26,133,616 | A | G | **-5.062** | 4.14E-07 | **-3.178** | **-6.356** | 12 |
| rs3857546 | 6 | 26,157,762 | T | C | **-6.049** | 1.46E-09 | **-3.941** | **-7.882** | 12 |
| rs7749823 | 6 | 26,158,079 | A | C | **5.817** | 6.01E-09 | **3.777** | **7.553** | 12 |
| rs10484439 | 6 | 26,309,908 | A | G | **-4.983** | 6.27E-07 | **-3.106** | **-6.212** | 13 |
| rs9366653 | 6 | 26,354,247 | A | G | **-4.798** | 1.60E-06 | **-2.910** | **-5.821** | 13 |
| rs9379851 | 6 | 26,354,780 | A | C | **4.838** | 1.31E-06 | **2.955** | **5.910** | 13 |
| rs9393705 | 6 | 26,361,011 | A | G | **-4.808** | 1.52E-06 | **-2.923** | **-5.846** | 13 |
| rs9393708 | 6 | 26,362,643 | T | C | **4.857** | 1.19E-06 | **2.978** | **5.957** | 13 |
| rs9358932 | 6 | 26,362,705 | T | C | **-4.909** | 9.17E-07 | **-3.034** | **-6.067** | 13 |
| rs9379855 | 6 | 26,364,930 | T | C | **4.881** | 1.06E-06 | **3.005** | **6.011** | 13 |
| rs9379856 | 6 | 26,366,836 | A | C | **4.790** | 1.66E-06 | **2.901** | **5.802** | 13 |
| rs9379858 | 6 | 26,367,689 | T | C | **4.832** | 1.35E-06 | **2.949** | **5.898** | 13 |
| rs9393710 | 6 | 26,367,833 | A | G | **4.853** | 1.21E-06 | **2.974** | **5.948** | 13 |
| rs9379859 | 6 | 26,369,549 | T | C | **-4.798** | 1.60E-06 | **-2.910** | **-5.821** | 13 |
| rs12176317 | 6 | 26,372,786 | A | G | **4.940** | 7.80E-07 | **3.064** | **6.128** | 13 |
| rs9393713 | 6 | 26,373,678 | A | G | **-4.864** | 1.15E-06 | **-2.986** | **-5.972** | 13 |
| rs9393714 | 6 | 26,373,740 | T | G | **-4.963** | 6.93E-07 | **-3.082** | **-6.165** | 13 |
| rs2073529 | 6 | 26,375,159 | A | G | **4.896** | 9.76E-07 | **3.021** | **6.043** | 13 |
| rs1977 | 6 | 26,377,546 | A | G | **4.947** | 7.54E-07 | **3.069** | **6.137** | 13 |
| rs1978 | 6 | 26,377,573 | A | T | **-5.036** | 4.76E-07 | **-3.149** | **-6.297** | 13 |
| rs2076030 | 6 | 26,426,856 | A | G | **-5.044** | 4.56E-07 | **-3.159** | **-6.318** | 13 |
| rs13195509 | 6 | 26,463,660 | A | G | **-5.036** | 4.77E-07 | **-3.149** | **-6.297** | 13 |
| rs3799380 | 6 | 26,467,182 | T | C | **5.018** | 5.23E-07 | **3.132** | **6.265** | 13 |
| rs3734544 | 6 | 26,468,660 | A | G | **-4.747** | 2.07E-06 | **-2.853** | **-5.706** | 13 |
| rs9366658 | 6 | 26,469,866 | A | G | **-4.748** | 2.05E-06 | **-2.855** | **-5.710** | 13 |
| rs9358944 | 6 | 26,469,875 | A | C | **4.757** | 1.96E-06 | **2.864** | **5.729** | 13 |
| rs9358945 | 6 | 26,472,114 | A | G | **4.730** | 2.25E-06 | **2.832** | **5.665** | 13 |
| rs7773938 | 6 | 26,474,044 | T | C | **-4.767** | 1.87E-06 | **-2.875** | **-5.749** | 13 |
| rs6456728 | 6 | 26,477,779 | A | G | **-5.010** | 5.46E-07 | **-3.123** | **-6.246** | 13 |
| rs9358946 | 6 | 26,478,927 | A | G | **-4.754** | 1.99E-06 | **-2.862** | **-5.724** | 13 |
| rs16891725 | 6 | 26,479,150 | T | C | **-5.135** | 2.82E-07 | **-3.243** | **-6.485** | 13 |
| rs7756567 | 6 | 26,481,642 | T | G | **4.759** | 1.94E-06 | **2.867** | **5.733** | 13 |
| rs2093169 | 6 | 26,495,099 | T | C | **-4.745** | 2.08E-06 | **-2.852** | **-5.704** | 13 |
| rs13198716 | 6 | 26,582,035 | T | C | **-5.450** | 5.05E-08 | **-3.505** | **-7.011** | 13 |
| rs6904071 | 6 | 27,047,256 | A | G | **-4.775** | 1.80E-06 | **-2.883** | **-5.766** | 14 |
| rs6913660 | 6 | 27,091,425 | A | C | **-4.806** | 1.54E-06 | **-2.921** | **-5.843** | 14 |
| rs13194053 | 6 | 27,143,883 | T | C | **4.762** | 1.92E-06 | **2.869** | **5.738** | 14 |
| rs13219354 | 6 | 27,185,664 | T | C | **4.892** | 1.00E-06 | **3.017** | **6.034** | 14 |
| rs3800307 | 6 | 27,185,792 | A | T | **-4.802** | 1.57E-06 | **-2.916** | **-5.833** | 14 |
| rs4452638 | 6 | 27,229,265 | A | G | **-4.913** | 8.96E-07 | **-3.039** | **-6.077** | 14 |
| rs6938200 | 6 | 27,231,150 | A | G | **4.787** | 1.69E-06 | **2.897** | **5.794** | 14 |
| rs6932590 | 6 | 27,248,931 | T | C | **5.115** | 3.14E-07 | **3.225** | **6.450** | 14 |
| rs3800316 | 6 | 27,256,102 | A | C | **5.653** | 1.57E-08 | **3.691** | **7.381** | 14 |
| rs7746199 | 6 | 27,261,324 | T | C | **-6.275** | 3.50E-10 | **-4.128** | **-8.257** | 14 |
| rs16897515 | 6 | 27,278,020 | A | C | **-5.783** | 7.34E-09 | **-3.773** | **-7.546** | 14 |
| rs6923811 | 6 | 27,289,776 | T | C | **4.929** | 8.28E-07 | **3.055** | **6.110** | 14 |
| rs13195040 | 6 | 27,413,924 | A | G | **5.379** | 7.49E-08 | **3.424** | **6.848** | 15 |
| rs7749305 | 6 | 27,446,566 | T | C | **5.490** | 4.03E-08 | **3.546** | **7.092** | 15 |
| rs6904596 | 6 | 27,491,299 | A | G | **-5.461** | 4.74E-08 | **-3.516** | **-7.033** | 15 |
| rs10484399 | 6 | 27,534,528 | A | G | **5.822** | 5.80E-09 | **3.777** | **7.553** | 15 |
| rs17749927 | 6 | 27,669,976 | A | G | **6.108** | 1.01E-09 | **3.988** | **7.977** | 16 |
| rs9295740 | 6 | 27,689,502 | A | G | **-5.340** | 9.30E-08 | **-3.394** | **-6.789** | 16 |
| rs2056925 | 6 | 27,690,905 | A | G | **4.916** | 8.85E-07 | **3.040** | **6.081** | 16 |
| rs17750424 | 6 | 27,701,122 | T | C | **6.045** | 1.49E-09 | **3.941** | **7.882** | 16 |
| rs17693963 | 6 | 27,710,165 | A | C | **6.399** | 1.56E-10 | **4.291** | **8.582** | 16 |
| rs6901520 | 6 | 27,714,575 | T | G | **-4.745** | 2.08E-06 | **-2.852** | **-5.704** | 16 |
| rs9283880 | 6 | 27,715,243 | A | C | **4.686** | 2.78E-06 | **2.775** | **5.550** | 16 |
| rs9461405 | 6 | 27,719,375 | T | G | **-4.841** | 1.29E-06 | **-2.959** | **-5.918** | 16 |
| rs7776351 | 6 | 27,726,731 | T | C | **-4.807** | 1.53E-06 | **-2.922** | **-5.845** | 16 |
| rs7759217 | 6 | 27,730,463 | A | G | **4.785** | 1.71E-06 | **2.895** | **5.790** | 16 |
| rs742046 | 6 | 27,739,254 | T | C | **4.937** | 7.91E-07 | **3.062** | **6.124** | 16 |
| rs2179095 | 6 | 27,750,858 | T | C | **-4.945** | 7.60E-07 | **-3.069** | **-6.137** | 16 |
| rs200483 | 6 | 27,774,824 | A | G | **-5.164** | 2.41E-07 | **-3.254** | **-6.509** | 16 |
| rs200484 | 6 | 27,775,674 | A | G | **5.044** | 4.56E-07 | **3.159** | **6.318** | 16 |
| rs370155 | 6 | 27,782,031 | T | G | **5.051** | 4.39E-07 | **3.166** | **6.332** | 16 |
| rs2747054 | 6 | 27,783,359 | A | G | **5.075** | 3.87E-07 | **3.187** | **6.373** | 16 |
| rs200501 | 6 | 27,788,942 | T | C | **-5.099** | 3.42E-07 | **-3.207** | **-6.415** | 16 |
| rs200490 | 6 | 27,796,935 | T | G | **-5.095** | 3.48E-07 | **-3.204** | **-6.409** | 16 |
| rs34706883 | 6 | 27,805,255 | A | C | **6.217** | 5.07E-10 | **4.061** | **8.123** | 16 |
| rs13212651 | 6 | 27,806,985 | A | G | **6.185** | 6.20E-10 | **4.048** | **8.095** | 16 |
| rs201002 | 6 | 27,808,192 | A | G | **5.143** | 2.71E-07 | **3.243** | **6.485** | 16 |
| rs175597 | 6 | 27,810,626 | T | C | **5.137** | 2.79E-07 | **3.243** | **6.485** | 16 |
| rs200995 | 6 | 27,813,694 | T | C | **5.146** | 2.66E-07 | **3.243** | **6.487** | 16 |
| rs200994 | 6 | 27,813,813 | T | G | **-4.668** | 3.05E-06 | **-2.750** | **-5.500** | 16 |
| rs7449460 | 6 | 27,814,677 | T | C | **5.103** | 3.35E-07 | **3.213** | **6.425** | 16 |
| rs200991 | 6 | 27,815,494 | A | C | **-5.122** | 3.02E-07 | **-3.231** | **-6.462** | 16 |
| rs13194781 | 6 | 27,815,639 | A | G | **6.206** | 5.45E-10 | **4.061** | **8.123** | 16 |
| rs200990 | 6 | 27,815,823 | T | G | **5.204** | 1.95E-07 | **3.277** | **6.554** | 16 |
| rs200989 | 6 | 27,816,442 | A | G | **5.187** | 2.14E-07 | **3.274** | **6.548** | 16 |
| rs13199772 | 6 | 27,834,085 | A | G | **6.165** | 7.05E-10 | **4.032** | **8.065** | 16 |
| rs200948 | 6 | 27,835,272 | T | C | **5.141** | 2.73E-07 | **3.243** | **6.485** | 16 |
| rs200953 | 6 | 27,837,267 | T | C | **5.130** | 2.89E-07 | **3.237** | **6.474** | 16 |
| rs200956 | 6 | 27,839,746 | T | C | **4.691** | 2.72E-06 | **2.781** | **5.562** | 16 |
| rs13218875 | 6 | 27,884,012 | T | C | **-5.727** | 1.02E-08 | **-3.728** | **-7.455** | 16 |
| rs156737 | 6 | 27,895,213 | A | G | **4.897** | 9.71E-07 | **3.022** | **6.044** | 16 |
| rs276371 | 6 | 27,910,708 | A | T | **5.135** | 2.82E-07 | **3.243** | **6.485** | 16 |
| rs156743 | 6 | 27,967,089 | T | C | **4.808** | 1.52E-06 | **2.923** | **5.846** | 17 |
| rs149971 | 6 | 27,982,152 | A | G | **-4.648** | 3.35E-06 | **-2.728** | **-5.455** | 17 |
| rs149990 | 6 | 27,998,258 | A | G | **-4.936** | 7.96E-07 | **-3.061** | **-6.123** | 17 |
| rs149943 | 6 | 28,002,388 | A | G | **-4.837** | 1.32E-06 | **-2.954** | **-5.907** | 17 |
| rs202906 | 6 | 28,011,652 | T | C | **4.993** | 5.95E-07 | **3.114** | **6.227** | 17 |
| rs13197574 | 6 | 28,060,239 | T | C | **5.879** | 4.13E-09 | **3.795** | **7.590** | 17 |
| rs9468287 | 6 | 28,079,741 | A | C | **-5.374** | 7.69E-08 | **-3.419** | **-6.837** | 17 |
| rs4713139 | 6 | 28,092,685 | T | C | **-5.412** | 6.24E-08 | **-3.460** | **-6.921** | 17 |
| rs4713140 | 6 | 28,097,193 | A | G | **-5.478** | 4.30E-08 | **-3.533** | **-7.066** | 17 |
| rs3757188 | 6 | 28,107,357 | T | C | **5.356** | 8.52E-08 | **3.407** | **6.814** | 17 |
| rs13205911 | 6 | 28,124,114 | T | C | **-5.788** | 7.11E-09 | **-3.777** | **-7.553** | 17 |
| rs9380064 | 6 | 28,143,118 | A | G | **5.430** | 5.63E-08 | **3.486** | **6.973** | 17 |
| rs735765 | 6 | 28,170,297 | A | G | **-5.549** | 2.88E-08 | **-3.606** | **-7.213** | 18 |
| rs9366717 | 6 | 28,191,057 | T | C | **-5.226** | 1.73E-07 | **-3.295** | **-6.590** | 18 |
| rs9468317 | 6 | 28,198,456 | T | C | **5.072** | 3.94E-07 | **3.185** | **6.369** | 18 |
| rs9380069 | 6 | 28,203,300 | A | G | **5.539** | 3.04E-08 | **3.600** | **7.199** | 18 |
| rs1150711 | 6 | 28,208,535 | T | C | **5.077** | 3.84E-07 | **3.187** | **6.373** | 18 |
| rs9295768 | 6 | 28,209,102 | A | G | **-5.002** | 5.67E-07 | **-3.120** | **-6.239** | 18 |
| rs967005 | 6 | 28,210,688 | T | C | **-5.115** | 3.15E-07 | **-3.225** | **-6.450** | 18 |
| rs17720293 | 6 | 28,214,698 | T | C | **-6.015** | 1.79E-09 | **-3.906** | **-7.813** | 18 |
| rs10456362 | 6 | 28,221,816 | A | G | **-5.818** | 5.96E-09 | **-3.777** | **-7.553** | 18 |
| rs1679709 | 6 | 28,228,342 | A | G | **-5.741** | 9.39E-09 | **-3.732** | **-7.464** | 18 |
| rs1778508 | 6 | 28,229,881 | T | C | **5.846** | 5.03E-09 | **3.778** | **7.556** | 18 |
| rs2799077 | 6 | 28,234,597 | T | C | **-5.844** | 5.09E-09 | **-3.778** | **-7.556** | 18 |
| rs11965538 | 6 | 28,239,915 | A | G | **-5.845** | 5.06E-09 | **-3.778** | **-7.556** | 18 |
| rs1778484 | 6 | 28,240,798 | A | G | **5.019** | 5.20E-07 | **3.133** | **6.266** | 18 |
| rs1419183 | 6 | 28,242,794 | A | C | **5.847** | 5.01E-09 | **3.778** | **7.556** | 18 |
| rs1150726 | 6 | 28,243,042 | A | G | **-5.025** | 5.04E-07 | **-3.138** | **-6.276** | 18 |
| rs1150724 | 6 | 28,250,236 | T | C | **-5.005** | 5.59E-07 | **-3.122** | **-6.245** | 18 |
| rs6901575 | 6 | 28,250,984 | A | G | **-6.075** | 1.24E-09 | **-3.952** | **-7.905** | 18 |
| rs13211507 | 6 | 28,257,377 | T | C | **5.790** | 7.05E-09 | **3.777** | **7.553** | 18 |
| rs1936365 | 6 | 28,268,452 | C | G | **-5.801** | 6.60E-09 | **-3.777** | **-7.553** | 18 |
| rs2281042 | 6 | 28,268,819 | A | C | **6.054** | 1.41E-09 | **3.941** | **7.882** | 18 |
| rs1997660 | 6 | 28,269,663 | A | G | **5.307** | 1.11E-07 | **3.361** | **6.721** | 18 |
| rs9468333 | 6 | 28,271,198 | T | C | **-5.191** | 2.09E-07 | **-3.275** | **-6.550** | 18 |
| rs853693 | 6 | 28,282,648 | A | G | **-5.223** | 1.76E-07 | **-3.294** | **-6.589** | 18 |
| rs853690 | 6 | 28,285,482 | A | G | **-5.197** | 2.03E-07 | **-3.275** | **-6.550** | 18 |
| rs853685 | 6 | 28,288,785 | T | C | **-5.979** | 2.25E-09 | **-3.881** | **-7.761** | 18 |
| rs707907 | 6 | 28,291,240 | A | G | **5.286** | 1.25E-07 | **3.336** | **6.673** | 18 |
| rs853683 | 6 | 28,295,039 | A | G | **5.266** | 1.40E-07 | **3.328** | **6.656** | 18 |
| rs853681 | 6 | 28,296,650 | A | C | **-5.808** | 6.33E-09 | **-3.777** | **-7.553** | 18 |
| rs853679 | 6 | 28,296,863 | A | C | **-5.989** | 2.11E-09 | **-3.887** | **-7.773** | 18 |
| rs853676 | 6 | 28,299,687 | T | C | **-5.941** | 2.83E-09 | **-3.858** | **-7.716** | 18 |
| rs17312661 | 6 | 28,300,336 | A | G | **4.915** | 8.89E-07 | **3.040** | **6.079** | 18 |
| rs7772827 | 6 | 28,301,143 | T | C | **4.905** | 9.32E-07 | **3.031** | **6.062** | 18 |
| rs1416920 | 6 | 28,302,784 | A | G | **5.087** | 3.63E-07 | **3.198** | **6.397** | 18 |
| rs9468344 | 6 | 28,309,569 | T | G | **5.121** | 3.04E-07 | **3.230** | **6.461** | 18 |
| rs6912584 | 6 | 28,309,590 | T | C | **5.449** | 5.05E-08 | **3.505** | **7.011** | 18 |
| rs12180820 | 6 | 28,316,478 | T | C | **-4.927** | 8.34E-07 | **-3.054** | **-6.107** | 18 |
| rs213237 | 6 | 28,323,938 | T | C | **4.744** | 2.09E-06 | **2.850** | **5.701** | 18 |
| rs6921919 | 6 | 28,325,201 | C | G | **5.437** | 5.42E-08 | **3.495** | **6.989** | 18 |
| rs6922111 | 6 | 28,325,308 | T | C | **-5.389** | 7.09E-08 | **-3.433** | **-6.867** | 18 |
| rs213230 | 6 | 28,330,264 | A | G | **5.096** | 3.47E-07 | **3.204** | **6.409** | 18 |
| rs213228 | 6 | 28,331,252 | A | C | **5.242** | 1.59E-07 | **3.306** | **6.613** | 18 |
| rs7773051 | 6 | 28,340,316 | A | G | **-5.233** | 1.66E-07 | **-3.298** | **-6.596** | 18 |
| rs7774981 | 6 | 28,346,910 | T | C | **5.460** | 4.75E-08 | **3.516** | **7.033** | 18 |
| rs1052215 | 6 | 28,348,158 | T | G | **-4.845** | 1.27E-06 | **-2.961** | **-5.923** | 18 |
| rs2531825 | 6 | 28,349,264 | T | C | **-4.791** | 1.66E-06 | **-2.902** | **-5.804** | 18 |
| rs13213152 | 6 | 28,349,698 | A | G | **5.899** | 3.66E-09 | **3.810** | **7.620** | 18 |
| rs3734563 | 6 | 28,349,725 | A | G | **5.198** | 2.02E-07 | **3.275** | **6.550** | 18 |
| rs7764722 | 6 | 28,354,533 | T | C | **-5.222** | 1.77E-07 | **-3.294** | **-6.589** | 18 |
| rs9461458 | 6 | 28,355,875 | T | C | **-5.209** | 1.90E-07 | **-3.282** | **-6.563** | 18 |
| rs1361385 | 6 | 28,358,320 | A | G | **5.179** | 2.23E-07 | **3.269** | **6.538** | 18 |
| rs16894060 | 6 | 28,362,471 | T | C | **5.224** | 1.75E-07 | **3.294** | **6.589** | 18 |
| rs2041230 | 6 | 28,365,515 | T | C | **5.248** | 1.53E-07 | **3.309** | **6.617** | 18 |
| rs2232423 | 6 | 28,366,151 | A | G | **5.999** | 1.98E-09 | **3.892** | **7.784** | 18 |
| rs4357130 | 6 | 28,367,683 | T | G | **5.269** | 1.37E-07 | **3.328** | **6.656** | 18 |
| rs9468370 | 6 | 28,368,940 | T | C | **-5.245** | 1.56E-07 | **-3.307** | **-6.613** | 18 |
| rs13196606 | 6 | 28,370,078 | A | G | **-5.532** | 3.17E-08 | **-3.594** | **-7.188** | 18 |
| rs6907950 | 6 | 28,370,246 | T | C | **-5.228** | 1.72E-07 | **-3.295** | **-6.590** | 18 |
| rs6908137 | 6 | 28,370,393 | A | C | **5.244** | 1.57E-07 | **3.307** | **6.613** | 18 |
| rs4254981 | 6 | 28,371,402 | A | C | **5.141** | 2.73E-07 | **3.243** | **6.485** | 18 |
| rs6928773 | 6 | 28,375,210 | T | C | **5.141** | 2.74E-07 | **3.243** | **6.485** | 18 |
| rs1124131 | 6 | 28,380,248 | A | C | **5.151** | 2.59E-07 | **3.248** | **6.495** | 18 |
| rs6899389 | 6 | 28,381,140 | A | C | **-5.128** | 2.93E-07 | **-3.235** | **-6.470** | 18 |
| rs1558205 | 6 | 28,382,262 | A | C | **-5.228** | 1.71E-07 | **-3.295** | **-6.590** | 18 |
| rs6922169 | 6 | 28,382,932 | A | G | **-5.193** | 2.06E-07 | **-3.275** | **-6.550** | 18 |
| rs16894091 | 6 | 28,390,137 | A | T | **-5.465** | 4.62E-08 | **-3.518** | **-7.036** | 18 |
| rs16894095 | 6 | 28,390,230 | T | C | **-5.329** | 9.87E-08 | **-3.387** | **-6.774** | 18 |
| rs3800328 | 6 | 28,390,843 | T | C | **-5.164** | 2.42E-07 | **-3.254** | **-6.509** | 18 |
| rs2859365 | 6 | 28,391,465 | A | G | **4.712** | 2.45E-06 | **2.808** | **5.616** | 18 |
| rs1339898 | 6 | 28,395,506 | T | C | **-5.559** | 2.71E-08 | **-3.616** | **-7.233** | 18 |
| rs7740351 | 6 | 28,399,412 | A | C | **5.136** | 2.80E-07 | **3.243** | **6.485** | 18 |
| rs16894106 | 6 | 28,400,339 | A | G | **-5.464** | 4.66E-08 | **-3.518** | **-7.036** | 18 |
| rs7766356 | 6 | 28,400,538 | T | C | **4.969** | 6.72E-07 | **3.089** | **6.178** | 18 |
| rs2071965 | 6 | 28,403,225 | T | C | **-5.138** | 2.78E-07 | **-3.243** | **-6.485** | 18 |
| rs13190937 | 6 | 28,411,244 | A | G | **-4.683** | 2.82E-06 | **-2.772** | **-5.544** | 18 |
| rs2531804 | 6 | 28,411,303 | A | G | **4.776** | 1.78E-06 | **2.884** | **5.769** | 18 |
| rs13215804 | 6 | 28,415,572 | A | G | **4.700** | 2.60E-06 | **2.792** | **5.584** | 18 |
| rs13215054 | 6 | 28,502,794 | A | C | **-4.987** | 6.14E-07 | **-3.108** | **-6.216** | 18 |
| rs17336532 | 6 | 28,543,264 | T | C | **-4.677** | 2.91E-06 | **-2.764** | **-5.528** | 18 |
| rs13194504 | 6 | 28,630,691 | A | G | **-5.858** | 4.67E-09 | **-3.778** | **-7.556** | 19 |
| rs6908726 | 6 | 28,671,343 | C | G | **5.151** | 2.59E-07 | **3.248** | **6.495** | 19 |
| rs7775835 | 6 | 28,678,357 | T | C | **-5.814** | 6.10E-09 | **-3.777** | **-7.553** | 19 |
| rs9468413 | 6 | 28,689,672 | A | C | **6.120** | 9.38E-10 | **3.993** | **7.986** | 19 |
| rs9393929 | 6 | 28,696,063 | A | C | **-5.321** | 1.03E-07 | **-3.379** | **-6.759** | 19 |
| rs6456834 | 6 | 28,700,352 | T | G | **-6.124** | 9.12E-10 | **-3.993** | **-7.986** | 19 |
| rs1233579 | 6 | 28,712,663 | A | G | **5.558** | 2.72E-08 | **3.616** | **7.233** | 19 |
| rs1233599 | 6 | 28,731,188 | T | G | **5.688** | 1.29E-08 | **3.710** | **7.421** | 19 |
| rs1233604 | 6 | 28,734,676 | A | G | **-5.672** | 1.41E-08 | **-3.702** | **-7.403** | 19 |
| rs1233619 | 6 | 28,745,452 | A | G | **-5.665** | 1.47E-08 | **-3.702** | **-7.403** | 19 |
| rs1311918 | 6 | 28,753,646 | T | C | **-5.796** | 6.81E-09 | **-3.777** | **-7.553** | 19 |
| rs7767099 | 6 | 28,768,698 | T | C | **-5.792** | 6.97E-09 | **-3.777** | **-7.553** | 19 |
| rs3131343 | 6 | 28,775,564 | A | G | **-5.734** | 9.80E-09 | **-3.728** | **-7.455** | 19 |
| rs4324798 | 6 | 28,776,117 | A | G | **-5.650** | 1.61E-08 | **-3.688** | **-7.376** | 19 |
| rs3118357 | 6 | 28,802,149 | A | G | **-5.729** | 1.01E-08 | **-3.728** | **-7.455** | 19 |
| rs9257248 | 6 | 28,803,291 | T | C | **-5.714** | 1.10E-08 | **-3.726** | **-7.453** | 19 |
| rs3132389 | 6 | 28,831,021 | T | G | **5.720** | 1.06E-08 | **3.726** | **7.453** | 19 |
| rs3132390 | 6 | 28,832,788 | A | G | **5.277** | 1.31E-07 | **3.331** | **6.663** | 19 |
| rs3118370 | 6 | 28,833,101 | T | G | **5.712** | 1.12E-08 | **3.726** | **7.453** | 19 |
| rs3132392 | 6 | 28,838,629 | T | C | **5.708** | 1.14E-08 | **3.724** | **7.448** | 19 |
| rs3135309 | 6 | 28,855,805 | A | C | **5.645** | 1.65E-08 | **3.687** | **7.373** | 19 |
| rs2230683 | 6 | 28,891,176 | T | C | **5.509** | 3.61E-08 | **3.569** | **7.137** | 19 |
| rs3118361 | 6 | 28,898,287 | T | C | **-5.623** | 1.87E-08 | **-3.665** | **-7.330** | 19 |
| rs3130895 | 6 | 28,905,791 | A | G | **-5.594** | 2.21E-08 | **-3.644** | **-7.287** | 19 |
| rs3131073 | 6 | 28,920,972 | A | G | **-5.571** | 2.53E-08 | **-3.624** | **-7.247** | 19 |
| rs3130845 | 6 | 28,923,367 | A | G | **5.543** | 2.98E-08 | **3.602** | **7.203** | 19 |
| rs3130837 | 6 | 28,948,092 | T | G | **-5.559** | 2.71E-08 | **-3.616** | **-7.233** | 19 |
| rs3129791 | 6 | 28,954,293 | A | G | **-5.625** | 1.85E-08 | **-3.666** | **-7.331** | 19 |
| rs3130893 | 6 | 28,980,707 | A | G | **5.626** | 1.85E-08 | **3.666** | **7.331** | 19 |
| rs3129788 | 6 | 29,057,639 | A | G | **-5.682** | 1.33E-08 | **-3.709** | **-7.418** | 19 |
| rs3130773 | 6 | 29,095,908 | A | G | **-5.679** | 1.35E-08 | **-3.708** | **-7.415** | 19 |
| rs3116830 | 6 | 29,167,575 | A | G | **-5.690** | 1.27E-08 | **-3.710** | **-7.421** | 20 |
| rs3117337 | 6 | 29,211,556 | A | T | **-5.603** | 2.11E-08 | **-3.648** | **-7.297** | 20 |
| rs3130827 | 6 | 29,230,683 | T | C | **-4.657** | 3.20E-06 | **-2.739** | **-5.478** | 20 |
| rs3130834 | 6 | 29,248,149 | T | C | **5.728** | 1.02E-08 | **3.728** | **7.455** | 20 |
| rs3117425 | 6 | 29,260,431 | T | C | **-5.332** | 9.70E-08 | **-3.389** | **-6.777** | 20 |
| rs3117439 | 6 | 29,266,483 | A | G | **-5.298** | 1.17E-07 | **-3.350** | **-6.700** | 20 |
| rs3117427 | 6 | 29,274,136 | T | C | **5.263** | 1.42E-07 | **3.325** | **6.650** | 20 |
| rs6930435 | 6 | 29,301,222 | A | G | **-4.878** | 1.07E-06 | **-3.002** | **-6.005** | 20 |
| rs1014258 | 6 | 29,310,681 | T | C | **-4.888** | 1.02E-06 | **-3.014** | **-6.028** | 20 |
| rs9257793 | 6 | 29,335,154 | T | C | **5.481** | 4.24E-08 | **3.535** | **7.069** | 20 |
| rs3749971 | 6 | 29,342,775 | A | G | **-5.151** | 2.59E-07 | **-3.248** | **-6.495** | 20 |
| rs9257805 | 6 | 29,346,329 | A | G | **5.373** | 7.75E-08 | **3.418** | **6.836** | 20 |
| rs9257809 | 6 | 29,356,331 | A | G | **5.187** | 2.14E-07 | **3.274** | **6.548** | 20 |
| rs442694 | 6 | 29,356,687 | T | G | **-5.306** | 1.12E-07 | **-3.361** | **-6.721** | 20 |
| rs429479 | 6 | 29,372,323 | A | G | **5.156** | 2.53E-07 | **3.249** | **6.499** | 20 |
| rs406511 | 6 | 29,376,385 | C | G | **5.056** | 4.28E-07 | **3.170** | **6.341** | 20 |
| rs1535039 | 6 | 29,411,432 | T | C | **5.276** | 1.32E-07 | **3.331** | **6.662** | 20 |
| rs2523443 | 6 | 29,415,464 | A | C | **-5.200** | 1.99E-07 | **-3.276** | **-6.552** | 20 |
| rs2746149 | 6 | 29,435,355 | T | C | **5.565** | 2.62E-08 | **3.618** | **7.236** | 20 |
| rs2746150 | 6 | 29,442,701 | T | C | **-5.366** | 8.06E-08 | **-3.415** | **-6.829** | 20 |
| rs17184100 | 6 | 29,454,816 | A | C | **5.016** | 5.29E-07 | **3.131** | **6.262** | 20 |
| rs1233493 | 6 | 29,458,241 | A | G | **5.523** | 3.33E-08 | **3.584** | **7.169** | 20 |
| rs1233491 | 6 | 29,461,730 | C | G | **-5.284** | 1.26E-07 | **-3.336** | **-6.672** | 20 |
| rs1233480 | 6 | 29,477,414 | T | C | **-5.238** | 1.63E-07 | **-3.302** | **-6.604** | 20 |
| rs1233478 | 6 | 29,477,821 | T | G | **-4.808** | 1.52E-06 | **-2.923** | **-5.846** | 20 |
| rs404240 | 6 | 29,523,957 | A | G | **5.397** | 6.78E-08 | **3.444** | **6.887** | 20 |
| rs1235162 | 6 | 29,537,224 | A | G | **5.519** | 3.41E-08 | **3.581** | **7.163** | 20 |
| rs1233396 | 6 | 29,546,799 | A | G | **-5.136** | 2.80E-07 | **-3.243** | **-6.485** | 20 |
| rs926552 | 6 | 29,548,089 | A | G | **-5.037** | 4.74E-07 | **-3.149** | **-6.297** | 20 |
| rs3115631 | 6 | 29,986,324 | A | T | **-5.668** | 1.45E-08 | **-3.702** | **-7.403** | 21 |
| rs8321 | 6 | 30,032,522 | A | C | **5.752** | 8.80E-09 | **3.741** | **7.481** | 21 |
| rs9261290 | 6 | 30,038,647 | T | C | **5.760** | 8.42E-09 | **3.743** | **7.486** | 21 |
| rs1541269 | 6 | 30,103,360 | A | G | **-4.773** | 1.82E-06 | **-2.882** | **-5.763** | 21 |
| rs2517645 | 6 | 30,122,623 | T | C | **4.648** | 3.35E-06 | **2.728** | **5.455** | 21 |
| rs2523735 | 6 | 30,122,657 | C | G | **5.246** | 1.56E-07 | **3.307** | **6.613** | 21 |
| rs1573296 | 6 | 30,127,805 | A | G | **5.647** | 1.64E-08 | **3.687** | **7.373** | 21 |
| rs929157 | 6 | 30,137,209 | A | G | **-5.201** | 1.98E-07 | **-3.276** | **-6.553** | 21 |
| rs2523729 | 6 | 30,137,387 | A | G | **-5.666** | 1.46E-08 | **-3.702** | **-7.403** | 21 |
| rs2106072 | 6 | 30,153,363 | A | G | **-5.684** | 1.32E-08 | **-3.709** | **-7.418** | 21 |
| rs2517618 | 6 | 30,158,127 | A | G | **-5.150** | 2.60E-07 | **-3.248** | **-6.495** | 21 |
| rs2517614 | 6 | 30,163,955 | A | G | **-6.439** | 1.20E-10 | **-4.304** | **-8.609** | 21 |
| rs2523722 | 6 | 30,165,273 | T | C | **-6.428** | 1.30E-10 | **-4.304** | **-8.609** | 21 |
| rs2523721 | 6 | 30,166,266 | T | C | **-6.480** | 9.20E-11 | **-4.308** | **-8.616** | 21 |
| rs2523719 | 6 | 30,168,319 | T | G | **-5.226** | 1.74E-07 | **-3.295** | **-6.590** | 21 |
| rs2517612 | 6 | 30,169,092 | A | G | **5.188** | 2.12E-07 | **3.274** | **6.548** | 21 |
| rs2517611 | 6 | 30,169,327 | A | G | **6.423** | 1.34E-10 | **4.304** | **8.609** | 21 |
| rs2517610 | 6 | 30,170,280 | A | G | **6.577** | 4.79E-11 | **4.391** | **8.783** | 21 |
| rs1117490 | 6 | 30,170,510 | T | C | **6.582** | 4.64E-11 | **4.391** | **8.783** | 21 |
| rs2844776 | 6 | 30,171,827 | T | C | **6.572** | 4.97E-11 | **4.391** | **8.783** | 21 |
| rs971570 | 6 | 30,172,513 | A | C | **6.568** | 5.11E-11 | **4.391** | **8.783** | 21 |
| rs2021722 | 6 | 30,174,131 | T | C | **-6.593** | 4.30E-11 | **-4.391** | **-8.783** | 21 |
| rs885912 | 6 | 30,174,633 | A | C | **-6.560** | 5.40E-11 | **-4.391** | **-8.783** | 21 |
| rs2188100 | 6 | 30,181,883 | A | G | **-5.187** | 2.14E-07 | **-3.274** | **-6.548** | 21 |
| rs885916 | 6 | 30,202,571 | T | C | **-5.127** | 2.94E-07 | **-3.235** | **-6.470** | 21 |
| rs2523742 | 6 | 30,204,399 | C | G | **5.192** | 2.08E-07 | **3.275** | **6.550** | 21 |
| rs2844773 | 6 | 30,207,495 | A | C | **-4.999** | 5.78E-07 | **-3.119** | **-6.238** | 21 |
| rs3094077 | 6 | 30,226,123 | T | C | **-4.910** | 9.11E-07 | **-3.034** | **-6.068** | 21 |
| rs2516708 | 6 | 30,228,721 | A | G | **-4.690** | 2.73E-06 | **-2.780** | **-5.560** | 21 |
| rs2844766 | 6 | 30,230,661 | T | C | **4.741** | 2.13E-06 | **2.847** | **5.694** | 21 |
| rs3094073 | 6 | 30,231,224 | A | G | **-4.958** | 7.13E-07 | **-3.077** | **-6.155** | 21 |
| rs3130401 | 6 | 30,231,273 | A | G | **4.946** | 7.59E-07 | **3.069** | **6.137** | 21 |
| rs3132659 | 6 | 30,231,330 | T | C | **4.925** | 8.46E-07 | **3.051** | **6.101** | 21 |
| rs3132658 | 6 | 30,231,666 | T | C | **4.950** | 7.42E-07 | **3.071** | **6.141** | 21 |
| rs3094071 | 6 | 30,231,768 | A | G | **-4.950** | 7.43E-07 | **-3.071** | **-6.141** | 21 |
| rs3130403 | 6 | 30,232,009 | T | G | **4.956** | 7.21E-07 | **3.077** | **6.153** | 21 |
| rs3130404 | 6 | 30,232,250 | A | G | **4.898** | 9.67E-07 | **3.022** | **6.045** | 21 |
| rs3094630 | 6 | 30,232,436 | A | G | **-4.917** | 8.77E-07 | **-3.042** | **-6.085** | 21 |
| rs3129701 | 6 | 30,232,672 | T | C | **-4.903** | 9.44E-07 | **-3.028** | **-6.057** | 21 |
| rs3129702 | 6 | 30,232,785 | A | C | **-4.908** | 9.21E-07 | **-3.034** | **-6.067** | 21 |
| rs3094629 | 6 | 30,232,953 | A | G | **-4.858** | 1.19E-06 | **-2.979** | **-5.957** | 21 |
| rs3129703 | 6 | 30,233,558 | A | C | **-4.908** | 9.22E-07 | **-3.034** | **-6.067** | 21 |
| rs3130405 | 6 | 30,234,152 | T | C | **-4.894** | 9.88E-07 | **-3.019** | **-6.038** | 21 |
| rs3129832 | 6 | 30,234,657 | A | G | **4.930** | 8.21E-07 | **3.055** | **6.110** | 21 |
| rs2844764 | 6 | 30,234,668 | A | T | **-4.705** | 2.53E-06 | **-2.799** | **-5.599** | 21 |
| rs3129704 | 6 | 30,234,700 | T | C | **4.856** | 1.20E-06 | **2.977** | **5.953** | 21 |
| rs3129705 | 6 | 30,234,721 | A | G | **-4.894** | 9.90E-07 | **-3.019** | **-6.038** | 21 |
| rs2844762 | 6 | 30,236,754 | T | C | **4.656** | 3.23E-06 | **2.737** | **5.475** | 21 |
| rs3130380 | 6 | 30,279,130 | A | G | **-5.694** | 1.24E-08 | **-3.710** | **-7.421** | 21 |
| rs3094064 | 6 | 30,296,253 | A | G | **-5.631** | 1.79E-08 | **-3.669** | **-7.338** | 21 |
| rs3129837 | 6 | 30,306,306 | A | G | **4.897** | 9.74E-07 | **3.021** | **6.043** | 21 |
| rs3132649 | 6 | 30,321,057 | A | G | **-5.945** | 2.76E-09 | **-3.858** | **-7.716** | 21 |
| rs3094061 | 6 | 30,321,189 | A | C | **5.754** | 8.69E-09 | **3.741** | **7.481** | 21 |
| rs3130374 | 6 | 30,321,336 | T | C | **-5.719** | 1.07E-08 | **-3.726** | **-7.453** | 21 |
| rs3094627 | 6 | 30,321,482 | T | C | **5.826** | 5.68E-09 | **3.777** | **7.553** | 21 |
| rs3130375 | 6 | 30,321,732 | A | C | **-5.818** | 5.95E-09 | **-3.777** | **-7.553** | 21 |
| rs3130377 | 6 | 30,323,393 | T | C | **-5.598** | 2.17E-08 | **-3.644** | **-7.287** | 21 |
| rs3130350 | 6 | 30,327,839 | T | G | **-5.609** | 2.03E-08 | **-3.655** | **-7.310** | 21 |
| rs3094622 | 6 | 30,327,952 | A | G | **5.616** | 1.96E-08 | **3.657** | **7.314** | 21 |
| rs3130352 | 6 | 30,328,357 | T | C | **-5.609** | 2.03E-08 | **-3.655** | **-7.310** | 21 |
| rs3094621 | 6 | 30,328,753 | T | C | **5.537** | 3.08E-08 | **3.600** | **7.199** | 21 |
| rs3094057 | 6 | 30,329,966 | A | G | **-5.492** | 3.96E-08 | **-3.548** | **-7.096** | 21 |
| rs3132647 | 6 | 30,330,737 | A | G | **5.572** | 2.52E-08 | **3.624** | **7.247** | 21 |
| rs3094054 | 6 | 30,333,505 | T | G | **-5.588** | 2.29E-08 | **-3.639** | **-7.278** | 21 |
| rs3129809 | 6 | 30,335,621 | A | G | **5.357** | 8.46E-08 | **3.407** | **6.814** | 21 |
| rs3129817 | 6 | 30,342,753 | A | G | **-5.286** | 1.25E-07 | **-3.336** | **-6.673** | 21 |
| rs3129818 | 6 | 30,342,966 | T | G | **-5.125** | 2.98E-07 | **-3.233** | **-6.465** | 21 |
| rs3129820 | 6 | 30,343,569 | A | G | **-5.218** | 1.81E-07 | **-3.291** | **-6.582** | 21 |
| rs3132631 | 6 | 30,344,645 | T | C | **-5.089** | 3.60E-07 | **-3.198** | **-6.397** | 21 |
| rs3132630 | 6 | 30,345,118 | T | C | **-5.131** | 2.89E-07 | **-3.237** | **-6.474** | 21 |
| rs3129822 | 6 | 30,346,208 | T | G | **-5.116** | 3.13E-07 | **-3.225** | **-6.450** | 21 |
| rs3132625 | 6 | 30,347,720 | A | G | **5.051** | 4.39E-07 | **3.166** | **6.332** | 21 |
| rs3130123 | 6 | 30,352,647 | A | G | **-5.158** | 2.50E-07 | **-3.249** | **-6.499** | 21 |
| rs3130126 | 6 | 30,353,739 | A | C | **-5.157** | 2.51E-07 | **-3.249** | **-6.499** | 21 |
| rs3094050 | 6 | 30,358,591 | A | G | **5.090** | 3.58E-07 | **3.199** | **6.397** | 21 |
| rs3094703 | 6 | 30,358,957 | T | C | **-5.066** | 4.05E-07 | **-3.183** | **-6.366** | 21 |
| rs3094035 | 6 | 30,363,136 | T | G | **-5.173** | 2.30E-07 | **-3.263** | **-6.527** | 21 |
| rs3094034 | 6 | 30,363,351 | A | T | **5.446** | 5.16E-08 | **3.502** | **7.004** | 21 |
| rs3130116 | 6 | 30,365,448 | T | C | **5.655** | 1.56E-08 | **3.691** | **7.381** | 21 |
| rs3130247 | 6 | 30,515,043 | T | C | **5.564** | 2.63E-08 | **3.618** | **7.236** | 22 |
| rs3132610 | 6 | 30,544,401 | A | G | **5.701** | 1.19E-08 | **3.717** | **7.434** | 22 |
| rs9262135 | 6 | 30,618,906 | A | G | **5.790** | 7.05E-09 | **3.777** | **7.553** | 23 |
| rs9262141 | 6 | 30,644,137 | T | C | **5.828** | 5.61E-09 | **3.777** | **7.553** | 23 |
| rs9262142 | 6 | 30,650,026 | A | G | **-5.886** | 3.95E-09 | **-3.799** | **-7.598** | 23 |
| rs9262143 | 6 | 30,652,781 | T | C | **-5.841** | 5.18E-09 | **-3.778** | **-7.556** | 23 |
| rs1064627 | 6 | 30,698,541 | A | G | **4.926** | 8.39E-07 | **3.053** | **6.105** | 23 |
| rs1059612 | 6 | 30,708,955 | T | C | **-5.725** | 1.03E-08 | **-3.728** | **-7.455** | 23 |
| rs3129973 | 6 | 30,721,143 | T | C | **-5.369** | 7.92E-08 | **-3.416** | **-6.832** | 23 |
| rs3095328 | 6 | 30,723,781 | A | G | **-5.438** | 5.39E-08 | **-3.495** | **-6.989** | 23 |
| rs3095326 | 6 | 30,725,841 | T | C | **-5.355** | 8.55E-08 | **-3.407** | **-6.814** | 23 |
| rs3095340 | 6 | 30,726,939 | A | C | **5.339** | 9.33E-08 | **3.394** | **6.789** | 23 |
| rs3095336 | 6 | 30,738,446 | A | G | **-5.191** | 2.09E-07 | **-3.275** | **-6.550** | 23 |
| rs3130673 | 6 | 30,746,519 | T | G | **-5.122** | 3.02E-07 | **-3.231** | **-6.462** | 23 |
| rs3129985 | 6 | 30,762,542 | T | C | **-5.196** | 2.03E-07 | **-3.275** | **-6.550** | 23 |
| rs3131060 | 6 | 30,763,291 | A | G | **-5.160** | 2.47E-07 | **-3.251** | **-6.501** | 23 |
| rs3129986 | 6 | 30,763,562 | T | C | **-4.830** | 1.37E-06 | **-2.946** | **-5.893** | 23 |
| rs3131064 | 6 | 30,763,893 | T | C | **4.683** | 2.82E-06 | **2.772** | **5.544** | 23 |
| rs3130641 | 6 | 30,764,081 | T | C | **-5.231** | 1.68E-07 | **-3.296** | **-6.592** | 23 |
| rs1264377 | 6 | 30,764,907 | A | G | **-4.889** | 1.01E-06 | **-3.015** | **-6.030** | 23 |
| rs1264361 | 6 | 30,777,498 | A | G | **5.266** | 1.40E-07 | **3.328** | **6.656** | 23 |
| rs886424 | 6 | 30,782,002 | T | C | **-5.273** | 1.34E-07 | **-3.330** | **-6.661** | 23 |
| rs1264353 | 6 | 30,787,762 | A | C | **-5.350** | 8.81E-08 | **-3.405** | **-6.811** | 23 |
| rs1264350 | 6 | 30,796,545 | T | C | **5.594** | 2.22E-08 | **3.644** | **7.287** | 23 |
| rs2535340 | 6 | 30,838,497 | T | C | **5.212** | 1.87E-07 | **3.283** | **6.566** | 23 |
| rs1264324 | 6 | 30,855,211 | T | G | **5.358** | 8.40E-08 | **3.407** | **6.814** | 23 |
| rs1264322 | 6 | 30,857,894 | A | G | **-5.330** | 9.82E-08 | **-3.387** | **-6.774** | 23 |
| rs886422 | 6 | 30,864,279 | T | C | **-5.364** | 8.15E-08 | **-3.413** | **-6.826** | 23 |
| rs1049633 | 6 | 30,867,527 | A | G | **-5.170** | 2.34E-07 | **-3.260** | **-6.521** | 23 |
| rs1264312 | 6 | 30,872,982 | A | G | **-5.341** | 9.26E-08 | **-3.394** | **-6.789** | 23 |
| rs886420 | 6 | 30,879,636 | T | C | **-5.286** | 1.25E-07 | **-3.336** | **-6.673** | 23 |
| rs1264308 | 6 | 30,879,987 | T | C | **-5.236** | 1.64E-07 | **-3.301** | **-6.601** | 23 |
| rs1264304 | 6 | 30,882,415 | T | C | **-5.226** | 1.73E-07 | **-3.295** | **-6.590** | 23 |
| rs3131921 | 6 | 30,907,335 | T | C | **5.749** | 8.99E-09 | **3.739** | **7.478** | 23 |
| rs3132581 | 6 | 30,913,458 | A | G | **-5.807** | 6.35E-09 | **-3.777** | **-7.553** | 23 |
| rs3130782 | 6 | 30,914,843 | T | C | **-5.716** | 1.09E-08 | **-3.726** | **-7.453** | 23 |
| rs3094086 | 6 | 30,919,391 | A | G | **-5.943** | 2.80E-09 | **-3.858** | **-7.716** | 23 |
| rs3132580 | 6 | 30,920,124 | A | G | **-5.919** | 3.24E-09 | **-3.833** | **-7.665** | 23 |
| rs3131934 | 6 | 30,931,844 | T | C | **5.557** | 2.74E-08 | **3.616** | **7.233** | 23 |
| rs3131783 | 6 | 30,932,068 | A | G | **-5.949** | 2.69E-09 | **-3.858** | **-7.716** | 23 |
| rs1634721 | 6 | 30,977,680 | A | G | **-5.286** | 1.25E-07 | **-3.336** | **-6.673** | 23 |
| rs3130544 | 6 | 31,058,340 | A | C | **-4.828** | 1.38E-06 | **-2.946** | **-5.891** | 24 |
| rs3130557 | 6 | 31,094,703 | T | C | **-5.008** | 5.51E-07 | **-3.123** | **-6.246** | 24 |
| rs1265099 | 6 | 31,105,413 | A | G | **4.659** | 3.18E-06 | **2.740** | **5.480** | 24 |
| rs7750641 | 6 | 31,129,310 | T | C | **-4.985** | 6.21E-07 | **-3.107** | **-6.213** | 24 |
| rs3132510 | 6 | 31,172,151 | T | C | **5.035** | 4.78E-07 | **3.148** | **6.297** | 24 |
| rs3134792 | 6 | 31,312,326 | T | G | **5.071** | 3.96E-07 | **3.185** | **6.369** | 25 |
| rs2596565 | 6 | 31,353,329 | A | G | **-5.162** | 2.44E-07 | **-3.253** | **-6.506** | 25 |
| rs3131618 | 6 | 31,434,621 | A | G | **5.103** | 3.35E-07 | **3.213** | **6.425** | 25 |
| rs3131643 | 6 | 31,442,782 | A | G | **-5.140** | 2.74E-07 | **-3.243** | **-6.485** | 25 |
| rs3099844 | 6 | 31,448,976 | A | C | **-4.985** | 6.19E-07 | **-3.107** | **-6.214** | 25 |
| rs3094011 | 6 | 31,451,836 | T | C | **4.935** | 8.00E-07 | **3.061** | **6.123** | 25 |
| rs3094005 | 6 | 31,465,047 | T | G | **-4.888** | 1.02E-06 | **-3.014** | **-6.028** | 25 |
| rs3101018 | 6 | 31,705,864 | T | C | **-4.756** | 1.98E-06 | **-2.864** | **-5.727** | 26 |
| rs3132445 | 6 | 31,712,196 | A | G | **-4.711** | 2.46E-06 | **-2.807** | **-5.614** | 26 |
| rs3130484 | 6 | 31,715,882 | T | C | **4.712** | 2.45E-06 | **2.808** | **5.616** | 26 |
| rs3131379 | 6 | 31,721,033 | A | G | **-4.707** | 2.52E-06 | **-2.801** | **-5.602** | 26 |
| rs3117574 | 6 | 31,725,230 | A | G | **-4.737** | 2.17E-06 | **-2.842** | **-5.684** | 26 |
| rs3131378 | 6 | 31,725,285 | A | G | **4.730** | 2.24E-06 | **2.832** | **5.665** | 26 |
| rs3117575 | 6 | 31,726,253 | T | C | **4.743** | 2.11E-06 | **2.849** | **5.699** | 26 |
| rs3117577 | 6 | 31,727,474 | A | G | **4.731** | 2.23E-06 | **2.834** | **5.667** | 26 |
| rs3115672 | 6 | 31,727,897 | T | C | **-4.758** | 1.96E-06 | **-2.865** | **-5.730** | 26 |
| rs707938 | 6 | 31,729,359 | A | G | **4.699** | 2.61E-06 | **2.791** | **5.582** | 26 |
| rs3115674 | 6 | 31,799,076 | T | G | **4.929** | 8.26E-07 | **3.055** | **6.110** | 26 |
| rs3130679 | 6 | 31,807,540 | A | G | **5.009** | 5.48E-07 | **3.123** | **6.246** | 26 |
| rs519417 | 6 | 31,878,433 | A | G | **-4.992** | 5.97E-07 | **-3.113** | **-6.226** | 26 |
| rs497309 | 6 | 31,892,484 | A | C | **4.964** | 6.90E-07 | **3.083** | **6.166** | 26 |
| rs1270942 | 6 | 31,918,860 | A | G | **5.026** | 5.00E-07 | **3.140** | **6.279** | 26 |
| rs389884 | 6 | 31,940,897 | A | G | **4.978** | 6.44E-07 | **3.099** | **6.199** | 26 |
| rs1150753 | 6 | 32,059,867 | A | G | **5.008** | 5.49E-07 | **3.123** | **6.246** | 26 |
| rs1150752 | 6 | 32,064,726 | T | C | **4.988** | 6.09E-07 | **3.109** | **6.219** | 26 |
| rs3130288 | 6 | 32,096,001 | A | C | **-4.848** | 1.24E-06 | **-2.967** | **-5.934** | 26 |
| rs3132971 | 6 | 32,230,256 | T | G | **4.957** | 7.16E-07 | **3.077** | **6.154** | 27 |
| rs7775397 | 6 | 32,261,252 | T | G | **4.939** | 7.84E-07 | **3.064** | **6.127** | 27 |
| rs9268219 | 6 | 32,284,108 | T | G | **4.950** | 7.44E-07 | **3.071** | **6.141** | 27 |
| rs9268235 | 6 | 32,290,208 | T | C | **-5.007** | 5.53E-07 | **-3.123** | **-6.246** | 27 |
| rs3117106 | 6 | 32,343,369 | T | C | **4.841** | 1.29E-06 | **2.959** | **5.918** | 27 |
| rs2894254 | 6 | 32,345,689 | T | G | **4.774** | 1.81E-06 | **2.882** | **5.763** | 27 |
| rs3135393 | 6 | 32,408,842 | A | G | **4.698** | 2.63E-06 | **2.790** | **5.580** | 27 |
| rs2239806 | 6 | 32,411,307 | T | C | **-4.674** | 2.95E-06 | **-2.759** | **-5.519** | 27 |
| rs3129890 | 6 | 32,414,273 | A | G | **5.243** | 1.58E-07 | **3.307** | **6.613** | 27 |
| rs9268856 | 6 | 32,429,719 | A | C | **-5.353** | 8.64E-08 | **-3.407** | **-6.814** | 27 |
| rs9268861 | 6 | 32,429,894 | A | C | **-4.716** | 2.40E-06 | **-2.813** | **-5.626** | 27 |
| rs9268862 | 6 | 32,430,167 | A | C | **5.322** | 1.03E-07 | **3.380** | **6.759** | 27 |
| rs7766843 | 6 | 32,430,729 | T | C | **-5.334** | 9.58E-08 | **-3.390** | **-6.781** | 27 |
| rs7746922 | 6 | 32,430,975 | A | C | **-4.894** | 9.90E-07 | **-3.019** | **-6.038** | 27 |
| rs9268878 | 6 | 32,431,292 | A | T | **-5.283** | 1.27E-07 | **-3.336** | **-6.672** | 27 |
| rs9268911 | 6 | 32,432,406 | A | G | **5.261** | 1.43E-07 | **3.324** | **6.648** | 27 |
| rs9268977 | 6 | 32,434,939 | T | C | **5.271** | 1.35E-07 | **3.330** | **6.659** | 27 |
| rs9268980 | 6 | 32,435,123 | A | G | **5.274** | 1.34E-07 | **3.330** | **6.661** | 27 |
| rs9269043 | 6 | 32,438,598 | T | C | **5.273** | 1.34E-07 | **3.330** | **6.661** | 27 |
| rs2157338 | 6 | 32,439,323 | T | C | **-5.267** | 1.38E-07 | **-3.328** | **-6.656** | 27 |
| rs2187823 | 6 | 32,439,508 | A | G | **-5.281** | 1.29E-07 | **-3.334** | **-6.668** | 27 |
| rs9270623 | 6 | 32,565,331 | A | C | **4.649** | 3.34E-06 | **2.728** | **5.456** | 27 |
| rs615672 | 6 | 32,574,171 | C | G | **-5.537** | 3.07E-08 | **-3.600** | **-7.199** | 27 |
| rs2858867 | 6 | 32,575,325 | A | G | **5.579** | 2.42E-08 | **3.630** | **7.260** | 27 |
| rs9272105 | 6 | 32,599,999 | A | G | **-5.690** | 1.27E-08 | **-3.710** | **-7.421** | 27 |
| rs9272143 | 6 | 32,600,803 | T | C | **4.946** | 7.59E-07 | **3.069** | **6.137** | 27 |
| rs9272219 | 6 | 32,602,269 | T | G | **-5.222** | 1.77E-07 | **-3.294** | **-6.589** | 27 |
| rs2187668 | 6 | 32,605,884 | T | C | **-5.087** | 3.63E-07 | **-3.198** | **-6.397** | 27 |
| rs9273012 | 6 | 32,611,641 | A | G | **5.386** | 7.20E-08 | **3.431** | **6.862** | 27 |
| rs9273327 | 6 | 32,623,223 | A | C | **5.062** | 4.14E-07 | **3.178** | **6.356** | 27 |
| rs2854275 | 6 | 32,628,428 | A | C | **-5.067** | 4.04E-07 | **-3.183** | **-6.366** | 27 |
| rs3129716 | 6 | 32,657,436 | T | C | **5.033** | 4.82E-07 | **3.147** | **6.294** | 27 |
| rs9276601 | 6 | 32,734,304 | A | G | **5.194** | 2.06E-07 | **3.275** | **6.550** | 27 |
| rs9276689 | 6 | 32,751,962 | T | C | **-5.382** | 7.38E-08 | **-3.426** | **-6.852** | 27 |
| rs7762279 | 6 | 32,755,290 | T | C | **5.522** | 3.35E-08 | **3.584** | **7.169** | 27 |
| rs1480380 | 6 | 32,913,246 | T | C | **-5.136** | 2.80E-07 | **-3.243** | **-6.485** | 28 |
| rs9276933 | 6 | 32,930,795 | T | C | **4.688** | 2.76E-06 | **2.777** | **5.553** | 28 |
| rs9462875 | 6 | 43,168,117 | A | G | **4.817** | 1.46E-06 | **2.933** | **5.867** | 29 |
| rs16896344 | 6 | 43,172,430 | A | G | **-4.739** | 2.15E-06 | **-2.845** | **-5.690** | 29 |
| rs10275045 | 7 | 1,920,826 | T | C | **-4.959** | 7.08E-07 | **-3.079** | **-6.158** | 30 |
| rs6952727 | 7 | 1,947,958 | A | G | **4.986** | 6.15E-07 | **3.108** | **6.216** | 30 |
| rs12537914 | 7 | 1,948,359 | T | C | **-4.873** | 1.10E-06 | **-2.996** | **-5.993** | 30 |
| rs4721184 | 7 | 1,950,784 | T | C | **4.833** | 1.35E-06 | **2.949** | **5.898** | 30 |
| rs11772205 | 7 | 1,951,236 | T | C | **-4.940** | 7.81E-07 | **-3.064** | **-6.128** | 30 |
| rs2056480 | 7 | 1,954,301 | A | G | **-4.930** | 8.24E-07 | **-3.055** | **-6.110** | 30 |
| rs4721190 | 7 | 1,954,732 | A | G | **-4.961** | 7.01E-07 | **-3.081** | **-6.162** | 30 |
| rs2280550 | 7 | 1,976,556 | A | G | **-4.908** | 9.19E-07 | **-3.034** | **-6.067** | 30 |
| rs12666575 | 7 | 2,004,421 | T | C | **-5.175** | 2.28E-07 | **-3.266** | **-6.531** | 30 |
| rs4721295 | 7 | 2,036,669 | T | G | **5.191** | 2.09E-07 | **3.275** | **6.550** | 30 |
| rs1107592 | 7 | 2,041,432 | A | G | **5.441** | 5.28E-08 | **3.498** | **6.995** | 30 |
| rs3800882 | 7 | 2,120,758 | A | G | **-4.857** | 1.19E-06 | **-2.978** | **-5.957** | 30 |
| rs3778969 | 7 | 2,139,990 | A | G | **-5.007** | 5.54E-07 | **-3.123** | **-6.246** | 30 |
| rs10224497 | 7 | 2,149,967 | A | G | **4.976** | 6.48E-07 | **3.098** | **6.197** | 30 |
| rs10239050 | 7 | 2,158,390 | A | G | **4.841** | 1.29E-06 | **2.959** | **5.918** | 30 |
| rs3800913 | 7 | 2,163,237 | A | G | **4.796** | 1.61E-06 | **2.909** | **5.818** | 30 |
| rs3800917 | 7 | 2,167,939 | A | G | **-5.186** | 2.15E-07 | **-3.273** | **-6.547** | 30 |
| rs3778991 | 7 | 2,172,455 | A | G | **-4.761** | 1.92E-06 | **-2.869** | **-5.737** | 30 |
| rs4721441 | 7 | 2,184,060 | T | C | **4.754** | 2.00E-06 | **2.862** | **5.723** | 30 |
| rs3779003 | 7 | 2,184,902 | T | C | **-4.768** | 1.86E-06 | **-2.875** | **-5.750** | 30 |
| rs10226475 | 7 | 2,226,162 | A | G | **5.449** | 5.06E-08 | **3.505** | **7.011** | 30 |
| rs4719457 | 7 | 2,230,225 | A | G | **5.156** | 2.53E-07 | **3.249** | **6.499** | 30 |
| rs10257990 | 7 | 2,232,671 | T | C | **5.000** | 5.73E-07 | **3.120** | **6.239** | 30 |
| rs3757440 | 7 | 2,272,936 | A | G | **4.659** | 3.18E-06 | **2.740** | **5.481** | 30 |
| rs7787274 | 7 | 2,275,993 | A | G | **-4.754** | 1.99E-06 | **-2.862** | **-5.724** | 30 |
| rs7799006 | 7 | 2,278,226 | T | C | **-4.744** | 2.10E-06 | **-2.850** | **-5.700** | 30 |
| rs33922585 | 7 | 2,285,178 | T | C | **-4.865** | 1.15E-06 | **-2.986** | **-5.972** | 30 |
| rs12534131 | 7 | 2,294,123 | T | C | **-4.809** | 1.52E-06 | **-2.923** | **-5.846** | 30 |
| rs10447541 | 7 | 2,301,578 | T | G | **4.782** | 1.74E-06 | **2.890** | **5.780** | 30 |
| rs11136729 | 8 | 4,177,791 | A | G | **4.878** | 1.07E-06 | **3.002** | **6.005** | 31 |
| rs10503253 | 8 | 4,180,844 | A | C | **5.077** | 3.84E-07 | **3.187** | **6.373** | 31 |
| rs10503256 | 8 | 4,214,179 | A | G | **5.203** | 1.96E-07 | **3.277** | **6.554** | 31 |
| rs1594353 | 8 | 4,225,233 | T | C | **4.841** | 1.29E-06 | **2.959** | **5.918** | 31 |
| rs11993860 | 8 | 4,228,083 | T | G | **4.650** | 3.31E-06 | **2.730** | **5.460** | 31 |
| rs12234997 | 8 | 9,237,708 | A | G | **4.667** | 3.06E-06 | **2.750** | **5.499** | 32 |
| rs821109 | 8 | 89,499,611 | A | G | **4.807** | 1.53E-06 | **2.922** | **5.845** | 33 |
| rs716881 | 8 | 89,516,441 | T | G | **-4.782** | 1.73E-06 | **-2.890** | **-5.781** | 33 |
| rs7826357 | 8 | 89,542,166 | T | C | **5.004** | 5.61E-07 | **3.122** | **6.244** | 33 |
| rs6990990 | 8 | 89,548,722 | T | C | **5.007** | 5.54E-07 | **3.123** | **6.246** | 33 |
| rs16884251 | 8 | 89,555,353 | T | C | **4.887** | 1.02E-06 | **3.013** | **6.027** | 33 |
| rs16884273 | 8 | 89,558,684 | T | C | **-4.961** | 7.01E-07 | **-3.081** | **-6.162** | 33 |
| rs35385383 | 8 | 89,566,464 | C | G | **-4.995** | 5.88E-07 | **-3.116** | **-6.232** | 33 |
| rs1352318 | 8 | 89,566,903 | A | G | **5.075** | 3.88E-07 | **3.187** | **6.373** | 33 |
| rs7819913 | 8 | 89,568,393 | T | G | **-4.984** | 6.24E-07 | **-3.107** | **-6.213** | 33 |
| rs1580508 | 8 | 89,570,460 | A | G | **5.052** | 4.38E-07 | **3.166** | **6.332** | 33 |
| rs7814164 | 8 | 89,573,045 | A | G | **-5.059** | 4.21E-07 | **-3.175** | **-6.349** | 33 |
| rs10504857 | 8 | 89,582,625 | A | G | **-5.202** | 1.97E-07 | **-3.277** | **-6.554** | 33 |
| rs7838490 | 8 | 89,585,048 | A | G | **5.180** | 2.21E-07 | **3.269** | **6.538** | 33 |
| rs4484741 | 8 | 89,605,536 | T | C | **-5.230** | 1.70E-07 | **-3.295** | **-6.590** | 33 |
| rs9969514 | 8 | 89,605,999 | T | C | **-5.210** | 1.89E-07 | **-3.282** | **-6.563** | 33 |
| rs4269585 | 8 | 89,608,690 | T | C | **-5.203** | 1.97E-07 | **-3.277** | **-6.554** | 33 |
| rs4534168 | 8 | 89,609,651 | A | G | **-5.117** | 3.11E-07 | **-3.226** | **-6.452** | 33 |
| rs13272330 | 8 | 89,615,855 | A | G | **4.961** | 7.02E-07 | **3.081** | **6.162** | 33 |
| rs6990941 | 8 | 89,644,431 | T | C | **-5.183** | 2.19E-07 | **-3.271** | **-6.542** | 33 |
| rs12334864 | 8 | 89,646,080 | T | C | **-5.075** | 3.86E-07 | **-3.187** | **-6.373** | 33 |
| rs13257525 | 8 | 89,677,161 | A | G | **-5.165** | 2.40E-07 | **-3.255** | **-6.510** | 33 |
| rs16886095 | 8 | 89,713,000 | A | G | **5.053** | 4.35E-07 | **3.166** | **6.332** | 33 |
| rs7833665 | 8 | 89,721,771 | T | C | **5.112** | 3.20E-07 | **3.223** | **6.446** | 33 |
| rs7829812 | 8 | 89,722,274 | A | G | **-5.094** | 3.50E-07 | **-3.204** | **-6.409** | 33 |
| rs7004633 | 8 | 89,760,311 | A | G | **-5.668** | 1.45E-08 | **-3.702** | **-7.403** | 33 |
| rs7005110 | 8 | 89,760,620 | A | G | **-5.613** | 1.99E-08 | **-3.655** | **-7.310** | 33 |
| rs6989651 | 8 | 89,760,929 | T | C | **4.662** | 3.14E-06 | **2.743** | **5.487** | 33 |
| rs12352353 | 9 | 4,743,341 | A | G | **4.974** | 6.57E-07 | **3.095** | **6.189** | 34 |
| rs12352385 | 9 | 4,743,477 | A | C | **4.936** | 7.99E-07 | **3.061** | **6.123** | 34 |
| rs396861 | 9 | 4,743,626 | A | G | **4.949** | 7.46E-07 | **3.070** | **6.141** | 34 |
| rs41441548 | 10 | 34,150,821 | A | G | **4.696** | 2.66E-06 | **2.787** | **5.574** | 35 |
| rs7096169 | 10 | 104,618,695 | A | G | **4.766** | 1.88E-06 | **2.873** | **5.747** | 36 |
| rs4917985 | 10 | 104,624,072 | A | G | **-4.767** | 1.87E-06 | **-2.875** | **-5.749** | 36 |
| rs4919691 | 10 | 104,624,475 | T | G | **4.826** | 1.40E-06 | **2.942** | **5.885** | 36 |
| rs7085104 | 10 | 104,628,873 | A | G | **4.679** | 2.88E-06 | **2.767** | **5.533** | 36 |
| rs11191438 | 10 | 104,637,864 | C | G | **-5.138** | 2.77E-07 | **-3.243** | **-6.485** | 36 |
| rs10786719 | 10 | 104,637,992 | A | G | **5.114** | 3.16E-07 | **3.225** | **6.450** | 36 |
| rs3740390 | 10 | 104,638,480 | T | C | **-5.127** | 2.94E-07 | **-3.235** | **-6.470** | 36 |
| rs11191454 | 10 | 104,660,004 | A | G | **5.279** | 1.30E-07 | **3.332** | **6.664** | 36 |
| rs10748835 | 10 | 104,660,256 | A | G | **-5.208** | 1.90E-07 | **-3.282** | **-6.563** | 36 |
| rs1046778 | 10 | 104,661,484 | T | C | **4.984** | 6.23E-07 | **3.107** | **6.213** | 36 |
| rs7897654 | 10 | 104,662,458 | T | C | **4.936** | 7.97E-07 | **3.061** | **6.123** | 36 |
| rs4532960 | 10 | 104,667,406 | T | C | **-5.168** | 2.37E-07 | **-3.257** | **-6.515** | 36 |
| rs12221064 | 10 | 104,677,126 | T | C | **-5.268** | 1.38E-07 | **-3.328** | **-6.656** | 36 |
| rs2297786 | 10 | 104,679,978 | T | C | **-5.179** | 2.23E-07 | **-3.269** | **-6.538** | 36 |
| rs12411886 | 10 | 104,685,299 | A | C | **-5.256** | 1.47E-07 | **-3.317** | **-6.634** | 36 |
| rs10748836 | 10 | 104,693,917 | A | G | **-5.158** | 2.49E-07 | **-3.249** | **-6.499** | 36 |
| rs12413409 | 10 | 104,719,096 | A | G | **-5.188** | 2.13E-07 | **-3.274** | **-6.548** | 36 |
| rs10509757 | 10 | 104,752,960 | A | G | **-5.108** | 3.25E-07 | **-3.219** | **-6.438** | 36 |
| rs10883817 | 10 | 104,755,431 | A | G | **-5.140** | 2.75E-07 | **-3.243** | **-6.485** | 36 |
| rs11191499 | 10 | 104,764,271 | T | C | **5.322** | 1.02E-07 | **3.380** | **6.759** | 36 |
| rs11191514 | 10 | 104,773,364 | T | C | **-5.352** | 8.71E-08 | **-3.407** | **-6.814** | 36 |
| rs7914558 | 10 | 104,775,908 | A | G | **-5.243** | 1.58E-07 | **-3.307** | **-6.613** | 36 |
| rs4917994 | 10 | 104,811,699 | T | C | **-5.024** | 5.07E-07 | **-3.137** | **-6.274** | 36 |
| rs10883824 | 10 | 104,812,897 | A | G | **5.048** | 4.46E-07 | **3.163** | **6.326** | 36 |
| rs2275271 | 10 | 104,814,162 | T | C | **5.049** | 4.44E-07 | **3.164** | **6.327** | 36 |
| rs1926034 | 10 | 104,829,102 | A | G | **-5.192** | 2.08E-07 | **-3.275** | **-6.550** | 36 |
| rs10883826 | 10 | 104,830,819 | A | G | **5.072** | 3.94E-07 | **3.185** | **6.369** | 36 |
| rs943036 | 10 | 104,836,047 | T | C | **5.151** | 2.59E-07 | **3.248** | **6.495** | 36 |
| rs2296569 | 10 | 104,836,511 | A | G | **-5.087** | 3.63E-07 | **-3.198** | **-6.397** | 36 |
| rs943035 | 10 | 104,839,152 | T | C | **5.030** | 4.91E-07 | **3.144** | **6.288** | 36 |
| rs7092200 | 10 | 104,844,872 | T | C | **4.819** | 1.44E-06 | **2.935** | **5.871** | 36 |
| rs11191548 | 10 | 104,846,178 | T | C | **5.407** | 6.39E-08 | **3.456** | **6.912** | 36 |
| rs8139 | 10 | 104,848,123 | A | G | **-5.069** | 4.01E-07 | **-3.183** | **-6.367** | 36 |
| rs3740387 | 10 | 104,849,468 | A | G | **-5.044** | 4.56E-07 | **-3.159** | **-6.318** | 36 |
| rs3736922 | 10 | 104,850,632 | A | G | **-4.773** | 1.82E-06 | **-2.882** | **-5.763** | 36 |
| rs17094683 | 10 | 104,851,301 | T | G | **-5.367** | 8.00E-08 | **-3.415** | **-6.829** | 36 |
| rs11191560 | 10 | 104,869,038 | T | C | **5.499** | 3.82E-08 | **3.556** | **7.112** | 36 |
| rs12413046 | 10 | 104,871,204 | A | G | **5.399** | 6.70E-08 | **3.445** | **6.890** | 36 |
| rs2066323 | 10 | 104,871,361 | A | G | **5.078** | 3.82E-07 | **3.187** | **6.373** | 36 |
| rs746293 | 10 | 104,897,254 | T | G | **-5.071** | 3.97E-07 | **-3.185** | **-6.369** | 36 |
| rs732998 | 10 | 104,897,901 | T | C | **5.336** | 9.50E-08 | **3.391** | **6.782** | 36 |
| rs12220375 | 10 | 104,901,491 | T | C | **5.372** | 7.80E-08 | **3.418** | **6.836** | 36 |
| rs11191580 | 10 | 104,906,211 | T | C | **5.593** | 2.23E-08 | **3.644** | **7.287** | 36 |
| rs3977751 | 10 | 104,920,232 | A | G | **5.072** | 3.94E-07 | **3.185** | **6.369** | 36 |
| rs4917996 | 10 | 104,925,829 | A | C | **5.135** | 2.81E-07 | **3.243** | **6.485** | 36 |
| rs11191593 | 10 | 104,939,215 | T | C | **5.352** | 8.68E-08 | **3.407** | **6.814** | 36 |
| rs7920251 | 10 | 104,950,197 | T | C | **-5.068** | 4.02E-07 | **-3.183** | **-6.367** | 36 |
| rs10748839 | 10 | 104,953,547 | T | C | **4.999** | 5.78E-07 | **3.119** | **6.238** | 36 |
| rs4307650 | 10 | 104,959,852 | A | C | **-4.823** | 1.42E-06 | **-2.939** | **-5.878** | 36 |
| rs11191732 | 10 | 105,331,761 | A | G | **5.177** | 2.26E-07 | **3.267** | **6.534** | 37 |
| rs1025641 | 10 | 128,307,192 | T | C | **4.929** | 8.28E-07 | **3.055** | **6.110** | 38 |
| rs4356203 | 11 | 17,160,148 | A | G | **-4.763** | 1.90E-06 | **-2.870** | **-5.741** | 39 |
| rs1941776 | 11 | 98,104,605 | T | C | **-4.819** | 1.44E-06 | **-2.935** | **-5.871** | 40 |
| rs2852034 | 11 | 98,112,493 | A | C | **-4.835** | 1.33E-06 | **-2.952** | **-5.904** | 40 |
| rs2848519 | 11 | 98,113,179 | C | G | **-4.844** | 1.28E-06 | **-2.961** | **-5.922** | 40 |
| rs2848547 | 11 | 98,123,504 | T | C | **-4.829** | 1.37E-06 | **-2.946** | **-5.891** | 40 |
| rs2509843 | 11 | 98,125,404 | A | G | **-4.872** | 1.10E-06 | **-2.996** | **-5.991** | 40 |
| rs671789 | 11 | 125,294,614 | A | C | **-4.770** | 1.84E-06 | **-2.878** | **-5.757** | 41 |
| rs1615640 | 11 | 125,308,076 | C | G | **-4.666** | 3.07E-06 | **-2.748** | **-5.497** | 41 |
| rs11220082 | 11 | 125,323,965 | T | C | **5.153** | 2.57E-07 | **3.248** | **6.495** | 41 |
| rs666160 | 11 | 125,326,615 | A | G | **4.953** | 7.30E-07 | **3.074** | **6.148** | 41 |
| rs548181 | 11 | 125,461,709 | A | G | **-5.547** | 2.91E-08 | **-3.605** | **-7.211** | 41 |
| rs540723 | 11 | 125,489,621 | A | G | **-5.252** | 1.51E-07 | **-3.312** | **-6.625** | 41 |
| rs540436 | 11 | 125,512,619 | T | C | **-5.220** | 1.79E-07 | **-3.292** | **-6.584** | 41 |
| rs12575533 | 11 | 125,594,258 | T | C | **4.920** | 8.67E-07 | **3.045** | **6.089** | 41 |
| rs2217034 | 11 | 125,613,572 | T | C | **5.139** | 2.76E-07 | **3.243** | **6.485** | 41 |
| rs1054997 | 11 | 125,618,813 | A | G | **-5.149** | 2.61E-07 | **-3.247** | **-6.495** | 41 |
| rs7972947 | 12 | 2,170,433 | A | C | **-4.941** | 7.77E-07 | **-3.064** | **-6.128** | 42 |
| rs1006737 | 12 | 2,345,295 | A | G | **4.860** | 1.17E-06 | **2.980** | **5.960** | 42 |
| rs2159100 | 12 | 2,346,393 | T | C | **4.818** | 1.45E-06 | **2.935** | **5.870** | 42 |
| rs4765905 | 12 | 2,349,584 | C | G | **4.914** | 8.92E-07 | **3.039** | **6.078** | 42 |
| rs10774035 | 12 | 2,368,674 | T | C | **4.765** | 1.89E-06 | **2.872** | **5.745** | 42 |
| rs10744560 | 12 | 2,387,099 | T | C | **4.752** | 2.02E-06 | **2.859** | **5.719** | 42 |
| rs12436216 | 14 | 35,809,414 | A | G | **-4.932** | 8.14E-07 | **-3.057** | **-6.114** | 43 |
| rs8003074 | 14 | 35,821,692 | A | C | **4.938** | 7.91E-07 | **3.062** | **6.124** | 43 |
| rs10135277 | 14 | 35,823,231 | T | C | **-5.022** | 5.11E-07 | **-3.136** | **-6.273** | 43 |
| rs1869901 | 15 | 40,595,627 | A | G | **-5.095** | 3.49E-07 | **-3.204** | **-6.409** | 44 |
| rs3784397 | 15 | 40,596,844 | T | C | **-4.902** | 9.50E-07 | **-3.027** | **-6.054** | 44 |
| rs4131791 | 18 | 52,747,871 | T | C | **-4.823** | 1.42E-06 | **-2.939** | **-5.878** | 45 |
| rs4309482 | 18 | 52,750,469 | A | G | **4.869** | 1.12E-06 | **2.992** | **5.984** | 45 |
| rs11874716 | 18 | 52,750,688 | T | G | **4.876** | 1.08E-06 | **3.001** | **6.002** | 45 |
| rs12966547 | 18 | 52,752,017 | A | G | **-4.891** | 1.00E-06 | **-3.017** | **-6.034** | 45 |
| rs9951150 | 18 | 52,821,124 | A | G | **-4.757** | 1.96E-06 | **-2.864** | **-5.729** | 45 |
| rs9646596 | 18 | 53,049,212 | A | G | **4.749** | 2.04E-06 | **2.856** | **5.712** | 46 |
| rs17594526 | 18 | 53,058,238 | T | C | **4.883** | 1.04E-06 | **3.008** | **6.016** | 46 |
| rs17594665 | 18 | 53,063,719 | A | G | **4.982** | 6.30E-07 | **3.105** | **6.210** | 46 |
| rs17594721 | 18 | 53,065,892 | A | G | **-4.871** | 1.11E-06 | **-2.995** | **-5.990** | 46 |
| rs11152369 | 18 | 53,066,328 | A | C | **-4.859** | 1.18E-06 | **-2.979** | **-5.958** | 46 |
| rs17509991 | 18 | 53,067,184 | A | G | **5.005** | 5.58E-07 | **3.122** | **6.245** | 46 |
| rs17512836 | 18 | 53,194,961 | T | C | **-5.584** | 2.35E-08 | **-3.635** | **-7.271** | 46 |
| rs17597926 | 18 | 53,205,938 | A | G | **5.476** | 4.35E-08 | **3.532** | **7.063** | 46 |
